# A Versatile Theranostic Platform for Colorectal Cancer Peritoneal Metastases: Real‐Time Tumor‐Tracking and Photothermal‐Enhanced Chemotherapy

**DOI:** 10.1002/advs.202102256

**Published:** 2021-08-16

**Authors:** Tao Sun, Guangping Zhang, Tingting Ning, Qinjun Chen, Yongchao Chu, Yifan Luo, Haoyu You, Boyu Su, Chao Li, Qin Guo, Chen Jiang

**Affiliations:** ^1^ Key Laboratory of Smart Drug Delivery (Ministry of Education) Minhang Hospital State Key Laboratory of Medical Neurobiology and MOE Frontiers Center for Brain Science Institutes of Brain Science Department of Pharmaceutics School of Pharmacy Fudan University 826 Zhangheng Road Shanghai 201203 China; ^2^ Shandong Province Key Laboratory of Medical Physics and Image Processing Technology School of Physics and Electronics and Institute of Materials and Clean Energy Shandong Normal University 1 University Road Jinan 250358 P. R. China

**Keywords:** colorectal cancer peritoneal metastases, hyperthermic intraperitoneal chemotherapy, photothermal, theranostic, tumor tracking

## Abstract

A versatile tumor‐targeting stimuli‐responsive theranostic platform for peritoneal metastases of colorectal cancer is proposed in this work for tumor tracking and photothermal‐enhanced chemotherapy. A quenched photosensitizer (“off” state) is developed and escorted into a tumor‐targeting oxaliplatin‐embedded micelle. Once reaching the tumor cell, the micelle is clasped to release free oxaliplatin, as well as the “off” photosensitizer, which is further activated (“turned‐on”) in the tumor reducing microenvironment to provide optical imaging and photothermal effect. The combined results from hyperthermia‐enhanced chemotherapy, deep penetration, perfused O_2_, and the leveraged GSH‐ROS imbalance in tumor cells are achieved for improved antitumor efficacy and reduced systematic toxicity.

## Graphical abstract

A versatile theranostic platform is developed for colorectal cancer peritoneal metastases, to realize real‐time tumor‐tracking and meanwhile photothermal‐enhanced chemotherapy.

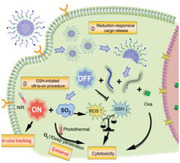

## 1 Introduction

Colorectal cancer is currently ranked among the top three morbidity cancers worldwide, accounting for ca. 2 million new cases every year.^[^ ^1^ ^]^ 25% of colorectal cancer patients will experience peritoneal metastases (CCPM) due to its natural of serosa infiltration, with a 5‐year survival rate less than 10% in clinic.^[^ ^2^ ^]^ CCPM, which is multiple and scattered, is the terminal stage of colorectal cancer, and the therapy is extremely difficult. CCPM are normally poorly vascularized, resulting a weak response to systemically‐administered chemotherapy.^[^ ^3^ ^]^

The gold standard treatment is hyperthermic intraperitoneal chemotherapy (HIPEC),^[^ ^4^ ^]^ which indicates a continuous perfused intraperitoneal circulation of heated chemotherapy fluid (≈43 °C) for ≈30–90 min post the invasive fistulation (Scheme S1, Supporting Information). HIPEC allows to intensify the direct and loco‐regional exposure of chemotherapeutics to CCPM, meanwhile the hyperthermia can enhance the chemotherapeutic efficacy to prolong the survival.^[^ ^5^ ^]^ Unfortunately, the cytotoxic drug is difficult to specifically and sufficiently accumulate into the metastatic nodules ascribed to the essential broad irrigation among enterocoelia during the short HIPEC session.^[^ ^6^ ^]^ The hyperthermia further exacerbates the damage to healthy tissues. 95% of CCPM patients post HIPEC were found with strong adverse reactions, including renal‐/hepato‐/gastrointestinal‐toxicity and myelosuppression.^[^ ^7^ ^]^ Besides the toxic burden from chemotherapy, HIPEC also brings about extra trauma to the patients. In order to establish the extracorporeal circulation, HIPEC requires complicated surgeries and sophisticated instruments. Patients receiving HIPEC often suffer from digestive fistula and peritonitis.^[^ ^8^ ^]^ Combined with the above, only 7% patients can receive a second HIPEC attempt,^[^ ^9^ ^]^ which greatly restricts the application. On the premise of maintaining the advantages of hyperthermia‐enhanced chemotherapy, new alternation with less trauma and tumor‐selective drug accumulation is in great demand.^[^ ^10^ ^]^

Photothermal strategy employs optical‐absorbing reagents to translate luminous energy into heat under the near‐infrared (NIR) light irradiation,^[^ ^11^ ^]^ which is highly accurate and strongly maneuverable.^[^ ^12^ ^]^ Thus, photo‐induced in‐situ mild hyperthermia (≈43 °C, Scheme S2, Supporting Information) could be an optimal alternation of fistulation‐based HIPEC. Once combined with chemotherapy into a single platform, photothermal‐assisted deep drug penetration is also greatly beneficial for the therapeutic outcomes.

Beyond the treatment, the early‐diagnosis and tumor‐tracking are crucial in establishing clinical pharmacotherapeutic schemes and assessing prognosis.^[^ ^13^ ^]^ In clinic, pathological biopsy of CCPM is hysteretic, while enhanced CT/MRI techniques are often interfered by nodule artifacts. Optical imaging holds great promise for tumor tracking due to its high sensitivity and safety, since FDA has approved near infrared (NIR) dye indocyanine green for image‐guided sentinel lymph node biopsy and surgical navigation of cancers in clinics.^[^ ^14^ ^]^ However, the “always‐on” type fluorophore sometimes lead to unrecognizable imaging between tumor and normal tissues with limited discrimination.^[^ ^15^ ^]^ Therefore, a “turn‐on” fluorescence probe, which can be specially activated in tumor microenvironment, is highly advantageous over current tactics.^[^ ^16^ ^]^

**Scheme 1:**
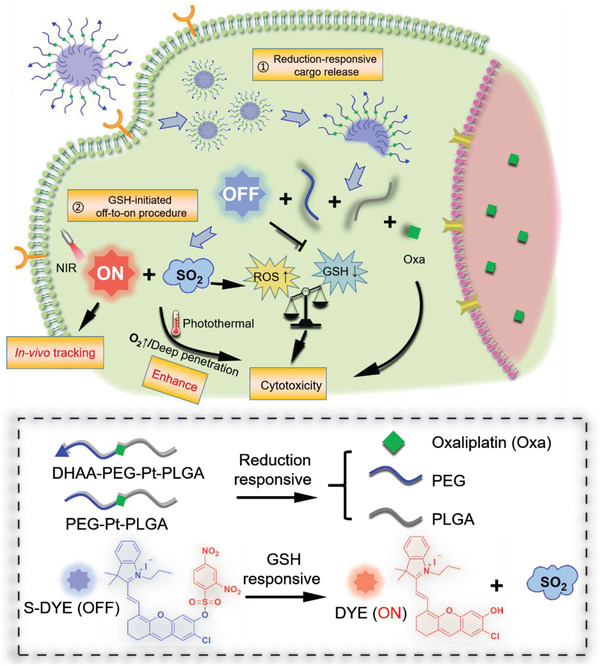
Illustration of the proposed strategy using a versatile theranostics platform for colorectal peritoneal metastases for tumor tracking and photothermal‐enhanced chemotherapy.

In this work as shown in **Scheme** **1**, we prepared a tumor‐targeting micelle from an oxaliplatin‐linked polymeric amphiphile (DHAA‐PEG‐Pt‐PLGA, DPPtP), which can encapsulate a quenched fluorophore (S‐DYE, SD). Once enrolled into the tumor reducing microenvironment, PEG‐Pt‐PLGA was cleaved to liberate the activated Oxa (II). The micelles then collapsed to release S‐DYE, which was further triggered with GSH to yield the on‐state DYE. Once irradiated with NIR light, the generated hyperthermia from DYE would in‐situ enhance the cytotoxicity of Oxa and deepen the drug penetration. Meanwhile, therapeutic SO_2_ would be simultaneously uncaged from the activation procedure of S‐DYE, to up‐regulate the ROS level. The intracellular consumption of GSH and improvement of ROS, and the adjunction of the photothermal‐enhanced chemotherapy, led to a synergistic antitumor efficacy.^[^ ^17^ ^]^ The strategy proposed in this work may provide an alternative approach for hyperthermia perfusion and CCPM treatment in clinic.

## 2 Results and Discussion

### 2.1 Design and Characterizations of the Photosensitizer

The satisfying properties of the designed activated photosensitizer should include an efficient turned‐on response, high sensitivity and selectivity, and favorable stability when in a quenched state. NIR fluorescent is advantageous over UV or visible light, since NIR light can cross deeper meanwhile causing limited injury to healthy tissues. The candidate serving for the aim proposed in this work should possess an off‐to‐on phenomenon regulated by the chemical modification with an electron‐attracting moiety or removal of the masking group. Meanwhile, both appropriate fluorescence quantum yield and photothermal conversion efficiency are required. The dinitrobenzene sulfonate group can act as a recognizing moiety to thiol‐containing spices which are at high levels in tumor cells. An NIR hemicyanine‐derived photosensitizer DYE quenched with a dinitrobenzene sulfonate group was finally selected (Scheme 1).

**Figure 1:**
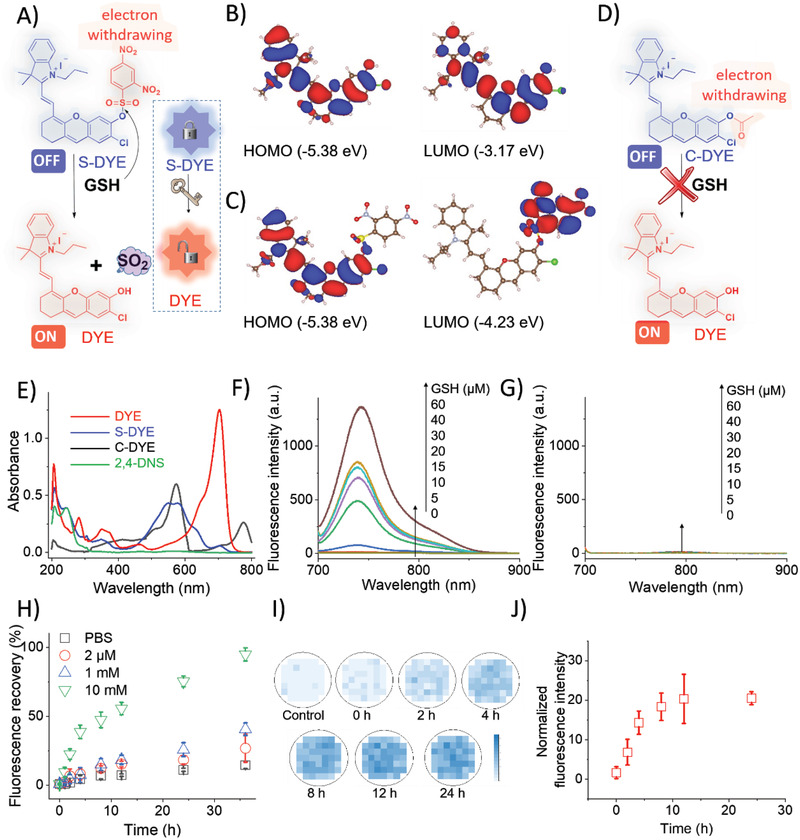
Design and characterizations of the photosensitizer. A) S‐DYE (off state) with an electron‐withdrawing moiety can respond to GSH to liberate free DYE (on state) and SO_2_; B) electron cloud and energy levels of the HOMO (−5.38 eV) and LUMO (−3.17 eV) of DYE; C) electron cloud and energy levels of the HOMO (−5.38 eV) and LUMO (−4.23 eV) of S‐DYE; D) C‐DYE (off state) with an ester electron‐withdrawing moiety cannot respond to GSH to liberate free DYE (on state); E) UV–vis absorbance of DYE, S‐DYE, C‐DYE, and 2,4‐DNS in ACN/water, respectively; F) the fluorescence variation of S‐DYE in presence of different concentrations of GSH; G) the fluorescence variation of C‐DYE in presence of different concentrations of GSH; H) the fluorescence recovery kinetics of S‐DYE over time in presence of different concentrations of GSH; I) the fluorescence recovery of S‐DYE over time at a cell level (HCT‐116) detecting by a plate reader; and J) the normalized fluorescence intensity variation of HCT‐116 cultured with S‐DYE detecting by a plate reader.

We synthesized a 2,4‐dinitrobenzenesulfonic ester (2,4‐DNS)‐silencing fluorophore (S‐DYE, SD, off state) from an NIR hemicyanine dye (belonging to ICG family, *λ*_ex/em_ = 679/730 nm). The “off‐to‐on” process can be initiated by the high level of glutathione (GSH) in tumor cells. Satisfactorily, the turned‐on DYE possesses both photothermal conversion efficiency (for mild hyperthermia) and fluorescence quantum yield (for tumor imaging). The parental NIR hemicyanine‐derived photosensitizer DYE was prepared following our previous work (Scheme S3, Supporting Information).^[^ ^18^ ^]^ DYE has been proved with fine anti‐photobleaching and anti‐photodegrading property. DYE exhibited a 28% fluorescence quantum yield (for NIR tracking) and 17% photothermal conversion efficiency (for generating hyperthermia). The fluorescence performance of DYE was effectively quenched by covalently attaching a 2,4‐DNS to the phenol group of DYE to yield S‐DYE. 2,4‐DNS is a typical electron‐withdrawing group that can induce the obvious deformation of the electronic cloud of the parental photosensitizer (**Figure** **1**A,B).^[^ ^19^ ^]^ To better understand the “on/off” mechanism, the computational study based on time‐dependent density functional theory (TD‐DFT) at the B3LYP/6‐31G(d) level using Gaussian 16 program^[^ ^20^ ^]^ was performed (Table S1, Supporting Information). The calculated results show that DYE has a strong absorbance at 535.6 nm and a strong emission at 662.2 nm (Table S2, Supporting Information). Meanwhile, S‐DYE also exhibited a strong absorbance at 532.5 nm, which is very close to that of DYE. However, the calculated emission of S‐DYE is at 1463.7 nm (Table S3, Supporting Information), which had a large red‐shift in comparison with that of DYE. This can explain why no fluorescence was observed for S‐DYE in the range of ≈700–900 nm. To explore the red‐shift of the fluorescence for S‐DYE, frontier molecular orbitals of the S1 state for DYE and S‐DYE have been plotted and shown in Figure 1B and 1C. The energy levels of the HOMO (−5.38 eV) and LUMO (−3.17 eV) of DYE were calculated in theory, were found varied greatly from the energy levels of the HOMO (−5.38 eV) and LUMO (−4.23 eV) of S‐DYE. It is clear that the spatial distribution and energy for the HOMO of S‐DYE is hardly affected by the 2,4‐DNS moiety. However, the spatial distribution of LUMO changed from DYE part to 2,4‐DNS block of S‐DYE due to the strong electron acceptors of the nitro groups. Meanwhile, the energy of LUMO for S‐DYE is lowered and thus the fluorescence of S‐DYE is red‐shifted and subsequently quenched at the initial NIR region.

In order to comprehensively learn the silencing essence and GSH‐responsiveness, an inert ethyl ester‐masking specie (C‐DYE) was synthesized, where the ethyl ester moiety is also an electron‐withdrawing group, but not detachable in presence with GSH (Figure 1D). The computational simulation results are in fine accordance with the spectroscopic discoveries, where both S‐DYE and C‐DYE were completely silenced at the NIR fluorescence region (Figure 1E). As predicted, the fluorescence‐recovery of S‐DYE exhibited fine GSH concentration‐dependent relationship (Figure 1F and Figures S17 and S18, Supporting Information), while C‐DYE remained irresponsive to GSH (Figure 1G). The combined theoretical and experimental results suggest the successful design of S‐DYE as a GSH‐initiated off‐to‐on probe.

The detailed procedure of S‐DYE**→**DYE was recorded by ^1^H NMR (Figure S19, Supporting Information), where an unambiguous substitution of GSH with the 2,4‐DNS block was tracked. The recovered DYE wouldn't be further reduced by GSH or destructed by the simultaneously released SO_2_ (Figure S20, Supporting Information), which was in accordance with the MS result (Figure S21, Supporting Information). DYE was found stable in presence of excess GSH. We further confirmed the remarkable GSH consuming capability by Ellman's Reagent during the S‐DYE→DYE reaction (Figure S22, Supporting Information).

We further studied the fluorescence‐recovery kinetics of S‐DYE in the presence of different GSH concentration, where a total recovery was completed after 36 h in presence of 10 mm GSH (mimicking the tumor's intracellular microenvironmental condition). Trace DYE reconversion was noticed in presence of GSH with lower concentration (1 mm mimicking extracellular condition, 2 mm mimicking plasma, and PBS 7.4). The NIR recovery (or DYE reconversion) from S‐DYE was also evaluated directly on a cell level. The fluorescence intensity gradually increased over time on human colorectal cells (HCT‐116), monitored (Figure 1I) and semi‐quantified (Figure 1H) by a plate reader.

### 2.2 Drug Formulation

Oxaliplatin (Oxa) is the first‐line therapeutic reagent in clinic for CCPM, however, direct exposal in the abdominal cavity may lead to serious damage to the healthy tissues. A case to illustrate this phenomenon could be the current preparation of an animal model for renal failure, which is a direct intraperitoneal injection with Oxa. The HIPEC duration is as short as ≈30–90 min, during when Oxa cannot selectively accumulate in CCPM nodules.

**Figure 2:**
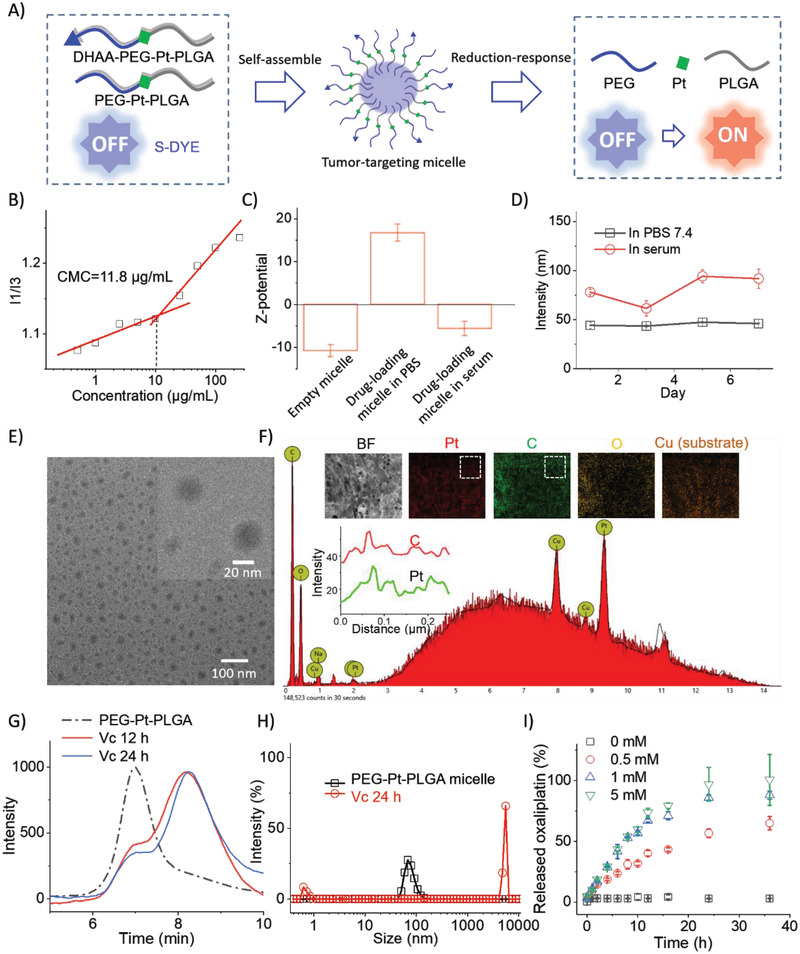
Drug formulation and reduction‐response characteristics. A) The composition of the tumor‐targeting micelle and corresponding reduction‐response characteristic, where Oxa‐release and “off‐to‐on” procedure of S‐DYE will occur upon meeting the reducing species; B) determination of the CMC value employing the pyrene fluorescence probe technique; C) the Z‐potentials of empty micelle, drug‐loading micelle in PBS, and drug‐loading micelle in serum; D) the size variation of the drug‐loading micelle in PBS or in serum upon placement at 4 °C over time; E) TEM image of the final tumor‐targeting micelle with a scale bar = 100 nm, plus a zoomed TEM image with a scale bar = 20 nm; F) the elemental analysis of the microaggregate on Cu plate employing EDS, where in the zoomed areas, C and Pt distribution are well in agreement; G) the molecular weight distribution of PPtP micelle before and after the Vc‐treatment, analyzed by GPC; H) the size distribution variation of PPtP micelle before and after Vc‐treatment with Vc by DLS; I) Oxa‐release kinetics from the PPtP micelle over time in presence of Vc with different concentrations at 37 °C.

We then employed oxidized Oxa (IV) as a linker to conjugate polyethylene glycol (PEG, 5K) and poly (lactic‐co‐glycolic acid (PLGA, 4,5K) together to form an amphiphilic building block PPtP for a micelle (Scheme S4, Supporting Information). A reaction between PEG‐NCO and Oxa‐OH was carried on to embed Oxa onto PEG to result PEG‐Pt. Then PLGA was linked to PEG‐Pt to obtain PPtP. The critical micelle concentration (CMC) value of PPtP was measured to be 11.8 µg mL^−1^ (**Figure** **2**B). Dehydroascorbic acid (DHAA), which can target tumors mediated by the accelerated transport rate of GLUT1 higher expressed on the tumor cells, was muddied to the ligand end to give DPPtP. DPPtP, PPtP, and S‐DYE could co‐assemble into well‐defined micelles (Figure 2A) with 45 nm diameter/+16.8 mV *ζ* potential in PBS and 81 nm diameter/−6.1 mV *ζ* potential in serum (Figure 2C,D) characterized with dynamic light scattering (DLS). The Z‐potential variation from negative to positive before and after drug‐loading was ascribed to the positive charge on the enveloped **S‐**DYE, while the Z‐potential variation from positive to negative upon being transferred into serum was more likely due to the adsorption with the protein corona in the serum (Figure 2C). The micelle show favorable stability in solution and serum upon placement at 4 °C in a week, with limited size variation. The encapsulation rate for S‐DYE into the micelle is 61.4% and the loading rate is 6.11% measured by HPLC. Spherical shape aggregates with ≈20–50 nm diameters were observed by transmission electron microscopy (TEM, Figure 2E). It is known that DLS measures the hydrated diameters of the micelles, while TEM manifests the solid spheres. The size increase from TEM to DLS results is due to the hydrated layers in the aqueous solution. The element‐distribution was undertaken by energy dispersive spectrometer (EDS, Figure 2F), and the Pt/C/O element distributions are found in fine agreement, suggesting the successful encapsulation of Oxa into the micelles.

In presence of Vitamin C (Vc, high level in tumor cells), PPtP could be cleaved upon the reduction of Oxa (IV) to liberate PEG and PLGA monitored by GPC, which could also be reflected by the apparent size variation detected by DLS (Figure 2H) and morphology change (Figure S23, Supporting Information). The Oxa linker enabled PPtP and corresponding micelles with reduction‐responsiveness capability. The Oxa‐release kinetics was further recorded with HPLC, where a clear Vc concentration‐dependence relationship was noticed. These combined results informed that the PPtP micelle could effectively encapsulate S‐DYE and release the cargo on‐demand under the reducing environment.

### 2.3 Cellular Uptake

**Figure 3:**
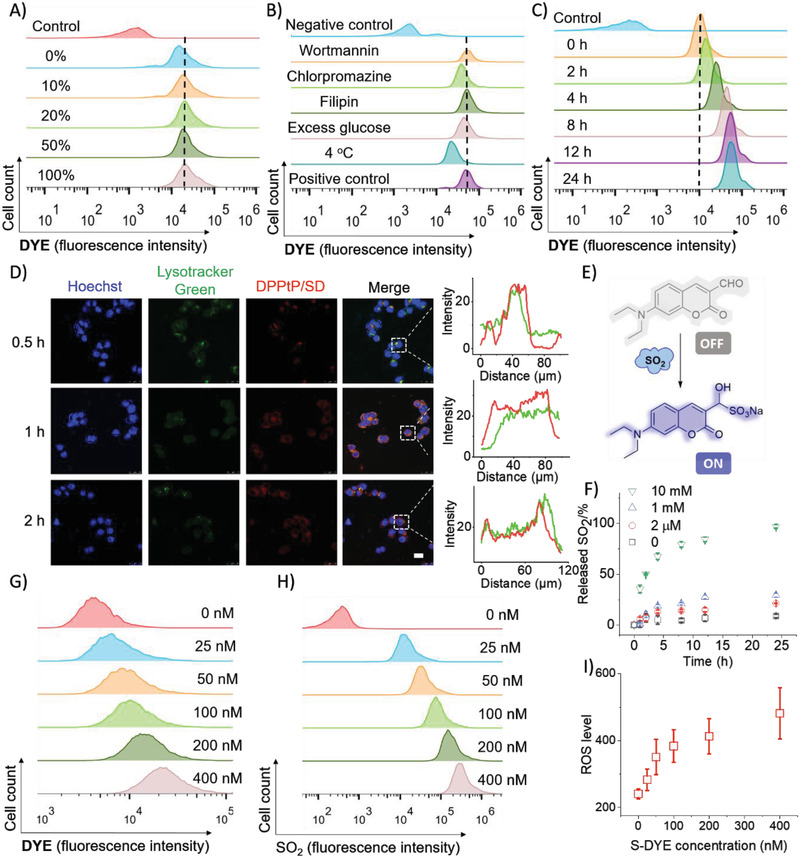
A) The determination of doping ratio of DPPtP among PPtP by flow cytometry; B) employing different inhibiting conditions (including wortmannin, chlorpromazine, filipin, excess glucose‐treatment, and 4 °C‐treatment) to determine the endocytosis pathways by flow cytometry; C) the florescence increase of HCT‐116 cells treated with DPPtP/SD by flow cytometry over time; D) confocal imaging of cellular uptake of DPPtP/SD after different incubation duration (scale bar = 5 µm) and corresponding semi‐quantitative results; E) the SO_2_‐consumption mechanism of the “turn‐on” type fluorescence SO_2_ probe; F) the SO_2_‐release kinetics from DPPtP/SD upon being treated with GSH with different concentrations; G) the NIR florescence increase of HCT‐116 cells treated with DPPtP/SD at different concentrations determined by flow cytometry; H) the SO_2_ increase of HCT‐116 cells treated with DPPtP/SD at different concentrations using the SO_2_‐probe determined by flow cytometry; and I) the ROS level increase on the HCT‐116 cells upon being treated with DPPtP/SD at different concentrations using the SO_2_‐probe determined by a plate‐reader.

The micelle composition was further optimized by doping with different percentage of DPPtP. Modification with DHAA (0 to 100 mw% doping ratio) did increase the cellular uptake of the micelles by tumor cells (**Figure** **3**A) detected by flow cytometer, which could be possibly mediated by the accelerated transporting rate through the GLUT1 transporters highly expressed on the tumor cells’ surface. There was no significant difference of the cellular uptake once the doping ratio reached over 20%, probably ascribed to the transporting saturation of GLUT1 to DHAA. It is know that too high doping ratio with the targeting ligand could increase the possibility of unexpected in vivo distribution, and 20% DPPtP among the micellar building blocks was finally chosen in the subsequent applications (referred as DPPtP). The internalization mechanism of the micelles into the tumor cells was further studied by being treated with different inhibitors to block possible cellular uptake routes (wortmannin [to block macropinocytosis], chlorpromazine [to block clathrin‐related pathway], filipin [to block caveolae‐mediated pathway], excess *D*‐glucose [GLUT1 competitor], and 4 °C‐treatment (to inhibit energy‐dependent pathway) (Figure 3B). The results suggested that clathrin‐related pathway is mainly responsive for the internalization, while GLUT1 participated in energy‐consuming actively recognizing and binding the micelles to the tumor cells.^[^ ^21^ ^]^

In succession, the intracellular behavior of the micelles was assessed. The tumor cells were treated with DPPtP/SD over different durations. A clear fluorescence intensity growth was noticed among the cells (Figure 3C), which was partially due to the increase of cellular uptake and partially due to the intracellular DYE reconversion (off‐to‐on). Inspired with this, the DPPtP/SD micelles were further used to treat HCT‐116 cells and monitored under confocal laser scanning microscopy to learn the essential cellular internalization (Figure 3D). Upon incubation for 0.5 h, the lysosomes were found co‐localized with the micelles, and the “off‐to‐on” step was already initiated, possibly due to the influence of the lysosomes’ reducing species. At 1 h, a lysosome‐escape phenomenon was observed to release the micelle into cytoplasm, where the “off‐to‐on” procedure was continued to lead an even fluorescence distribution among the cytoplasm. The serial lightening step was probably triggered by GSH that is rich in cytoplasm. At 2 h post the incubation, it was found that the “off‐to‐on” process was nearly completed on a cell level. The semi‐quantified results are also in agreement with the above micelles’ internalization pathway and intracellular progressive occurrence of the S‐DYE→DYE activation (Figure 3D). A clear internalization transfer into the cells mediated via lysosome pathway into cytoplasm, accompanied with the S‐DYE→DYE activation initiated by the encountered reducing species, was illustrated.

### 2.4 SO_2_ Release

Increasing evidences suggest that SO_2_ can be employed as a star specie in antitumor applications.^[^ ^22^ ^]^ SO_2_ was regarded as a new member in disturbing the intracellular imbalance by depleting GSH and inducing ROS generation upon being introduced into the tumor cells.^[^ ^23^ ^]^ GSH and ROS are both with high levels, and also playing the subtle balance within the tumor cells, which is considered important in maintaining tumors’ malignancy and invasiveness.^[^ ^24^ ^]^ However, SO_2_ is a typical gas molecule and possesses a relatively short half‐life in vivo,^[^ ^25^ ^]^ which greatly hinders the continuous exposure of SO_2_ in the tumor tissues. Solidifying gaseous SO_2_ into tumor‐targeted drug delivery system, and realizing an on‐demand release, are still with great importance but challenging.

In this theranostic system, we proposed during the S‐DYE→DYE reconversion, SO_2_ would be simultaneously and equivalently released (as evidenced by ^1^H NMR spectra in Figure S19, Supporting Information)^[^ ^26^ ^]^ through an electron transfer mechanism (FigureS20, Supporting Information), where S‐DYE was initially degraded by the thiol group of GSH via a thiol‐induced substitution of the 2,4‐DNS group anchored on Ar‐OH, thereby resulting the GSH‐2,4‐DNS conjugate. In addition, the secondary cleavage of 2,4‐DNS group would lead to the formation of DYE with free Ar‐OH, accompanied with the equivalent liberation of SO_2_.

In order to quantitatively learn the SO_2_‐release kinetics, we then synthesized an “off‐to‐on” type SO_2_ probe (Figure 3E). Nearly all SO_2_ would be released during 24 h in presence of 10 mm GSH, which was basically in accordance with the S‐DYE→DYE kinetics as shown in Figure 1H. The NIR florescence increase was found positively related to the SO_2_ level determined by flow cytometry (Figure 3G,H), when HCT‐116 cells were treated with DPPtP/SD at different concentrations. The higher the S‐DYE concentration was, the more GSH‐consumption and the higher of the ROS level would be (Figure 3I and Figure S25A, Supporting Information). In addition, as reported, the generated SO_2_ could also deplete the encountered GSH, and an increase in the ROS level. The combined and cascaded tailoring would obviously disturb the intratumoral GSH‐ROS balance to induce the apoptosis.^[^ ^27^ ^]^ Based on these in vitro experiments, we also detected the TNF‐*α* expression (reflecting ROS damage) of the tumors isolated from the orthotopic tumor‐bearing mice post different treatment by Western–Blots method. The results indicated that targeted SO_2_ delivery plus a photothermal treatment would lead to the highest ROS injury to the tumor cells (Figure S25B, Supporting Information).

### 2.5 In Vivo Distribution

**Figure 4:**
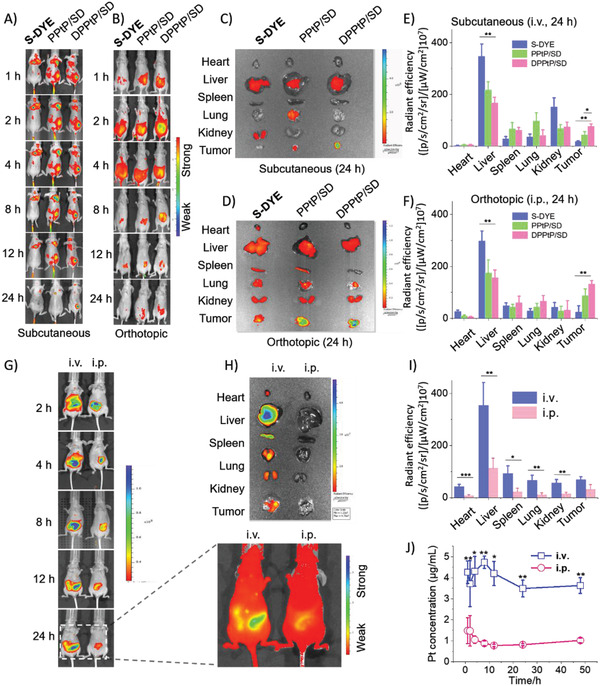
The in vivo distribution and “turned‐on” procedure of S‐DYE, PPtP/SD, and PPtP/SD over time on the A) subcutaneous (i.v.) and B) orthotopic (i.p.) tumor‐bearing mice observed by IVIS; the dissected main organs (from the top: heart, liver, spleen, lung, kidney, and collected tumor of the C) subcutaneous (i.v.) and D) orthotopic tumor‐bearing mice (i.p.) at different intervals post injection with S‐DYE, PPtP/SD, and DPPtP/SD over time observed under IVIS; semi‐quantification results of the distribution of the dissected main organs from the E) subcutaneous and F) orthotopic tumor‐bearing mice at 24 h post injection with S‐DYE, PPtP/SD, and DPPtP/SD observed under IVIS (*p < 0.05, **p < 0.01, or ***p < 0.001, *n* = 3); the in vivo distribution and “turned‐on” procedure of PPtP/SD over time on the orthotopic tumor‐bearing mice (i.p.) observed by IVIS; G) the orthotopic tumor‐bearing mice (i.v. and i.p. administration) at different intervals post injection with DPPtP/SD over time observed under IVIS (the contrast ratio of the enlarged image was adjusted to better illustrate the in vivo distribution and accumulation; the dissected main organs and collected tumor of the orthotopic tumor‐bearing mice (i.v. and i.p.) at different intervals post injection with DPPtP/SD over time H) observed under IVIS and I) corresponding semi‐quantification results at 24 h (*p < 0.05, **p < 0.01 or ***p < 0.01, *n* = 3); and J) the serum Pt concentration over time on the orthotopic tumor‐bearing mice (i.v. and i.p.) measured by ICP‐AES.

The fine tumor‐targeting in vivo and increased accumulation in the tumor sites are the premise and key feature for improving the cancer therapy efficiency.^[^ ^28^ ^]^ Subsequently, the in vivo distribution and activation of the theranostic system were studied in detail. In order to comprehensively assess the tumor‐targeting characteristics of the designed micelle, we constructed subcutaneous and orthotopic tumor‐bearing mice models. Free S‐DYE, PPtP/SD, and DPPtP/SD were intravenously injected into the subcutaneous tumor‐bearing mice via the tail vain (i.v.). It was noticed that most free S‐DYE would be cleared in 24 h, PPtP/SD and DPPtP/SD exhibited apparently increased tumor‐targeting and accumulation capability into the tumor site over time (**Figure** **4**A,C,E). Compared with PPtP/SD, DPPtP/SD shows a prolonged continuous accumulation into the tumor, and less liver/kidney distribution. On the orthotopic tumor‐bearing mice, free S‐DYE, PPtP/SD, and DPPtP/SD were intraperitoneally injected (i.p.). Again, DPPtP/SD displayed higher tumor‐targeting and accumulation capability (Figure 4B,D,F). These evidences imply that DPPtP/SD could apparently target to tumor and increase the tumor accumulation in vivo.

For better clinical translation, the polymers (PEG and PLGA) for the micellar building blocks are biodegradable materials approved by FDA.^[^ ^29^ ^]^ The hydrophilic PEG moiety outside the surface can prolong the systemic circulation by minimizing the immunological surveillance. Besides classic EPR effect (passive targeting), DHAA decorated onto the micelle could specifically recognize tumor cells via GLUT1 (active targeting), which is a member belonging to glucose transporter family overexpressed on many tumor cells. Furthermore, unlike glucose, DHAA‐transporting through the tumor cell membrane is a “single‐way” continuous accumulation.^[^ ^30^ ^]^ The self‐assembled micelle can thus effectively escort and directionally deliver the loaded cargos into tumors.

Essentially, S‐DYE was in “off” state and invisible under IVIS in vivo. Like the micelles’ internalization pathway shown in Figure 3D, the in vivo “come‐in”/“off‐to‐on” procedures that occurred in tumors are also progressive and parallel events. Based on the results in Figure 4A–F, clear information on “when”, “where”, and “how much” of the drug‐release in a non‐invasive and real‐time way could also be given, which could further provide useful directions on tumor‐tracking, prodrug design, drug fate study, rational clinical application, and therapeutic efficacy prediction.

Then, we attempted different administrating routes on the orthotopic tumor‐bearing mice models. Compared with i.v., administration through i.p. with the same amount of drug could display a more loco‐regional distribution (Figure 4G,H). Interestingly, on the premise of maintaining a reasonable drug concentration in the tumor (−53.6%), i.p. administration could drastically reduce the distribution among main organs (heart: −81.6%; liver: −67.8%; spleen: −75.3%; lung: −83.6%; kidney: −75.4%). We further investigated the pharmacokinetics for i.v./i.p. administrations, where the blood concentration of Pt via i.v. maintained at ≈3–4 times higher than the group via i.p. (Figure 4J). Plasma peritoneal barrier greatly restricts the specie exchange among blood and peritoneal, and the beneficence from i.v. administration in treating CCPM is limited. Due to the intrinsic existence of peritoneal‐vascular barrier, i.p. administration could remarkably minimize the systematic drug distribution and toxicity to the main organs.

### 2.6 Tumor Tracking

Current clinical diagnostic methods cannot identify and track CCPM due to its natural of scattering multiplicity on the peritoneum.^[^ ^31^ ^]^ For instance, MRI technique, as the gold standards of tumor diagnosis, is often interfered by nodule artifacts to result a total 40% precision.^[^ ^32^ ^]^ Meanwhile, optical imaging technique is a real‐time, non‐radiant and non‐invasive approach to monitor the onset and progression of tumors for the in‐situ longitudinal monitoring of molecular events.^[^ ^33^ ^]^ Upon being combined with the tumor‐targeting strategy, optical imaging technique can further offer relatively high spatiotemporal resolution, as well as superb sensitivity.^[^ ^34^ ^]^ By measuring the subtle changes of tumor biomarkers at a molecular level via the stimulus‐responsiveness of the “off‐to‐on” probes.^[^ ^35^ ^]^ However, real‐time probes and in vivo optical imaging of CCPM has yet to be demonstrated.^[^ ^36^ ^]^

**Figure 5:**
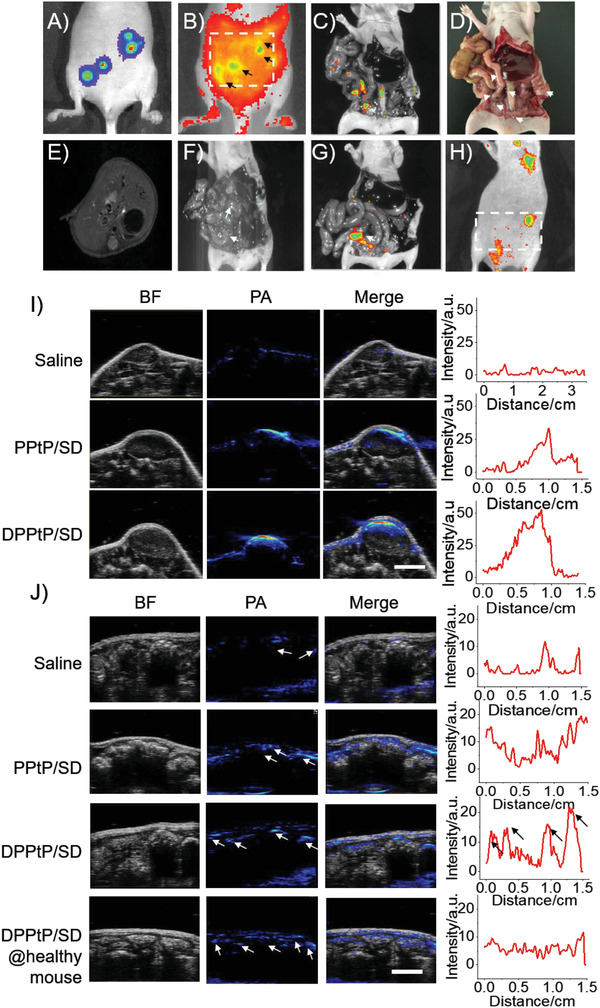
Tumor tracking of the orthotopic colorectal peritoneal metastases employing the versatile theranostics platform. A) Bioluminescence of the orthotopic tumor‐bearing mice exhibiting the precise tumor niche location; B) fluorescence tracking of the orthotopic tumor‐bearing mice 12 h post injection with DPPtP/SD (i.p.) with arrows indicating the niche and C) corresponding anatomical findings observed by IVIS; D) bright field of the anatomical appearance of the orthotopic tumor‐bearing mice; E) MRI diagnosis of the orthotopic tumor‐bearing mice; anatomical findings of the orthotopic tumor‐bearing mice 12 h post injection with F) S‐DYE (i.p.) and G) PPtP/SD (i.p.) observed with IVIS; H) fluorescence imaging of the healthy mice 12 h post injection with DPPtP/SD (i.p.) observed by IVIS; I) employing PA to observe the subcutaneous (i.v.) tumor‐bearing mice 12 h post injection with saline, PPtP/SD, and DPPtP/SD and corresponding semi‐quantification results of the PA signals (scale bar = 0.5 cm); J) employing PA to observe the orthotopic (i.p.) tumor‐bearing mice 12 h post injection with saline, PPtP/SD, and DPPtP/SD, plus the group of healthy mouse 12 h post injection with DPPtP/SD and corresponding semi‐quantification results of the PA signals (scale bar = 0.5 cm).

Based on the targeting/accumulating performance and “off‐to‐on” capability of DPPtP/SD,^[^ ^37^ ^]^ tracking of CCPM using NIR fluorescence were attempted. CT‐26‐luci cell line was used to construct an orthotopic tumor‐bearing mice model exhibiting the precise tumor nodule location by bioluminescence (**Figure** **5**A).^[^ ^38^ ^]^ After a 12 h post injection with DPPtP/SD (i.p.), distinct nodules were found under IVIS (Figure 5B), which are in fine accordance with the bioluminescence‐indicating niches. The anatomical (Figure 5C) and bright‐field (Figure 5D) results further support the tumor tracking faculty. In contrast, MRI could only provide blurry cicatrices at the already‐known niches (Figure 5E and Figure S26, Supporting Information). S‐DYE (i.p.) and PPtP/SD (i.p.) were also attempted with lower fluorescence intensity observed with IVIS (Figure 5G). DPPtP/SD (i.p.) was also administrated on the healthy mice, where unexpected and tanglesome distribution was monitored under IVIS (Figure 5H). 12 h post the administration, the “turn‐on” procedure from S‐DYE to DYE could also occur on healthy mice.

Moreover, we employed photoacoustic imaging (PA) to observe the subcutaneous (i.v.) (Figure 5I) and the orthotopic (i.p.) (Figure 5J) tumor‐bearing mice 12 h post injection with saline, PPtP/SD, and DPPtP/SD. Based on the semi‐quantification results of the PA signals, DPPtP/SD show the most advantageous signal to noise ratio (SNR), suggesting DPPtP/SD can also be employed as a tumor tracking strategy. Besides the above, 3D‐FLECT technique could also give useful information to indicate the niche (Figure S27, Supporting Information). We then launched a synergetic tumor to verdict (fluorescence+PA) on the randomly grouped healthy and CCPM mice in double‐blind trials, and the precision can reach as high as 70.6% (Table S4, Supporting Information). The dual‐mode imaging‐guided tumor tracking capability from this system would be applicable in directing the subsequent photothermal hyperthermia treatment.^[^ ^39^ ^]^

Informative optical bio‐imaging, especially NIR fluorescence, can provide useful non‐invasiveness and signals to confirm and quantify the specific analytes without radiation pollution. Photoacoustic bio‐imaging is another powerful in vivo tool that can transform the absorbed light into an ultrasound signal. An activable fluorescence/photoacoustic probe that can selectively leverage bio‐information into visual and monitoring readout. As to the oncologists, multiplexed detecting approaches are essentially important, since tumors are heterogeneous and complex. Multiplexed information from activable fluorescence/photoacoustic signals can lead to more precise diagnostics.

However, imaging‐guided photothermal therapy is still too far based on this work, given the fact that CCPM is multiple and scattered among the peritoneal. The subsequent photothermal treatment should be wide among the abdomen, which is beneficial in killing the tumor cells scattered among the peritoneal and delivered into the ascites

### 2.7 Photothermal Efficiency

Traditionally, PTT normally generates hyperthermia higher than 50 °C for inducing tumor ablation, which, however, can result in heat injury to normal organs nearby.^[^ ^40^ ^]^ Recently, a low‐temperature PTT (≈43 °C, LTPTT) was developed for effective apoptosis of tumor cells meanwhile with limited influence onto neighboring tissues.

**Figure 6:**
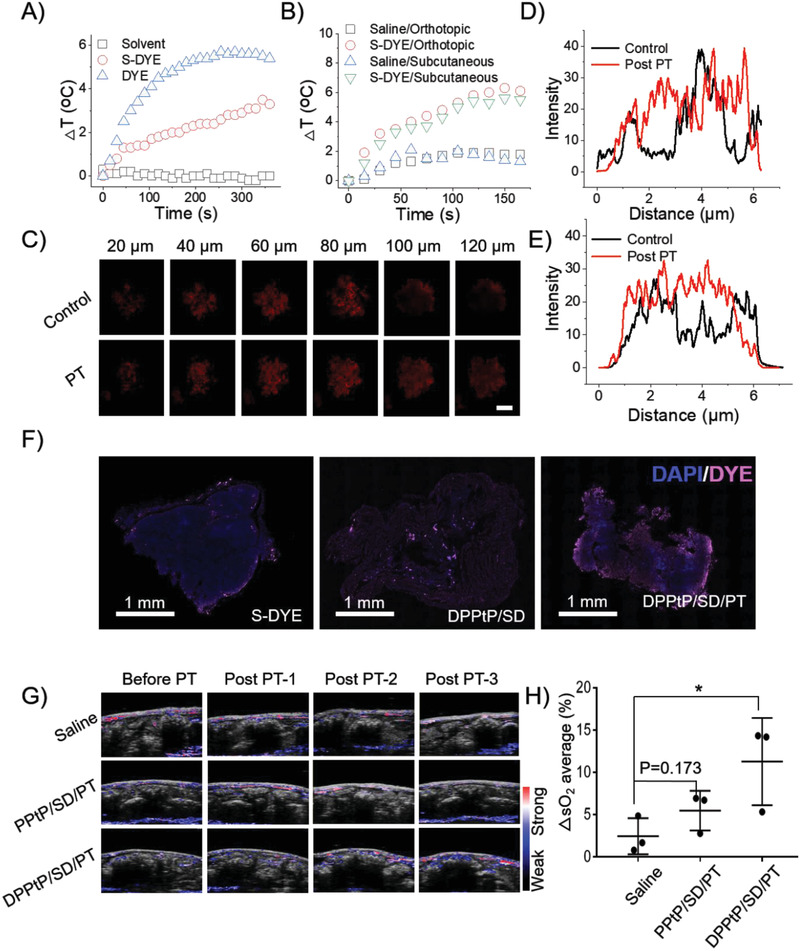
Photothermal‐assisted deep penetration. A) The temperature rise of different species (including blank, free S‐DYE, and DYE in ACN/PBS 7.4 [1:1, v:v]) upon a 680‐nm laser irradiation (0.3 W cm^−2^); B) the temperature rise of different species upon a 680‐nm laser irradiation (0.3 W cm^−2^) on orthotopic (i.p. with DPPtP/SD) or subcutaneous (i.v. with DPPtP/SD) tumor‐bearing mice; C) photothermal‐assisted deep penetration evaluated on tumor sphere (scale bar = 100 µm); representative semi‐quantitative results of the cross sections of D) 60 and E) 80 µm; F) photothermal‐assisted deep penetration and distribution evaluated on the orthotopic abdominal metastatic tumors; G) the blood oxygen content from three representative transections upon a 680‐nm laser irradiation (0.3 W cm^−2^, 5 min) and corresponding H) semi‐quantitative results measured by photoacoustic imaging (*p < 0.05, *n* = 3).

In this work, LTPTT strategy was innovatively introduced into the hyperthermia‐enhanced chemotherapy for CCPM. By modification with 2,4‐DNS moiety to tailor the spectroscopic property in the NIR region, S‐DYE shows a much slower heating curve than DYE under the same irradiation condition (**Figure** **6**A and Figure S28, Supporting Information). Compared with saline, a 6° increase at the tumor sites was found among both subcutaneous (i.v.) and orthotopic (i.p.) tumor‐bearing mice 12 h post administered with DPPtP/SD (Figure 6B). In addition, the photothermal‐assisted deep penetration was evaluated on the tumor sphere model, and the representative semi‐quantitative results of the cross sections of 60/80 µm demonstrated an evident deep penetration upon being treated with only 0.3 W cm^−2^ irradiation (Figure 6C–E). Similar deeper penetration was also found in the excised tumors (Figure 6F), which contributes to the enhancement of antitumor efficacy of therapeutics.^[^ ^41^ ^]^

Low O_2_ level in the hypoxic tumors significantly limits the antitumor efficacy of these ROS‐requiring anticancer modalities.^[^ ^42^ ^]^ Increasing data demonstrate that hypoxia is the major contributor to the poor prognosis of many anticancer therapies.^[^ ^43^ ^]^ Ascribed to the intrinsic existence of a peritoneal‐vascular barrier, CCPM represents a typical hypoxia tumor. More O_2_ supply in tumors is highly beneficial remitting the tumor hypoxia and potentiating the therapeutic outcomes.^[^ ^44^ ^]^ The abdominal blood O_2_ content 12 h post administered with DPPtP/SD and a 680‐nm laser irradiation (0.3 W cm^−2^, 5 min) was also increased (Figure 6G,H, saline: 2.5%, PPtP/SD/PT: 5.5%, and DPPtP/SD/PT: 11.3%) measured by PA. Similar O_2_‐increase results were also confirmed on the subcutaneous model (Figure S29, Supporting Information). Based on the combined performance shown above, DPPtP/SD is thus believed capable of fulfilling the requirements of hyperthermia‐enhanced chemotherapy.

### 2.8 Antitumor Efficacy

**Figure 7:**
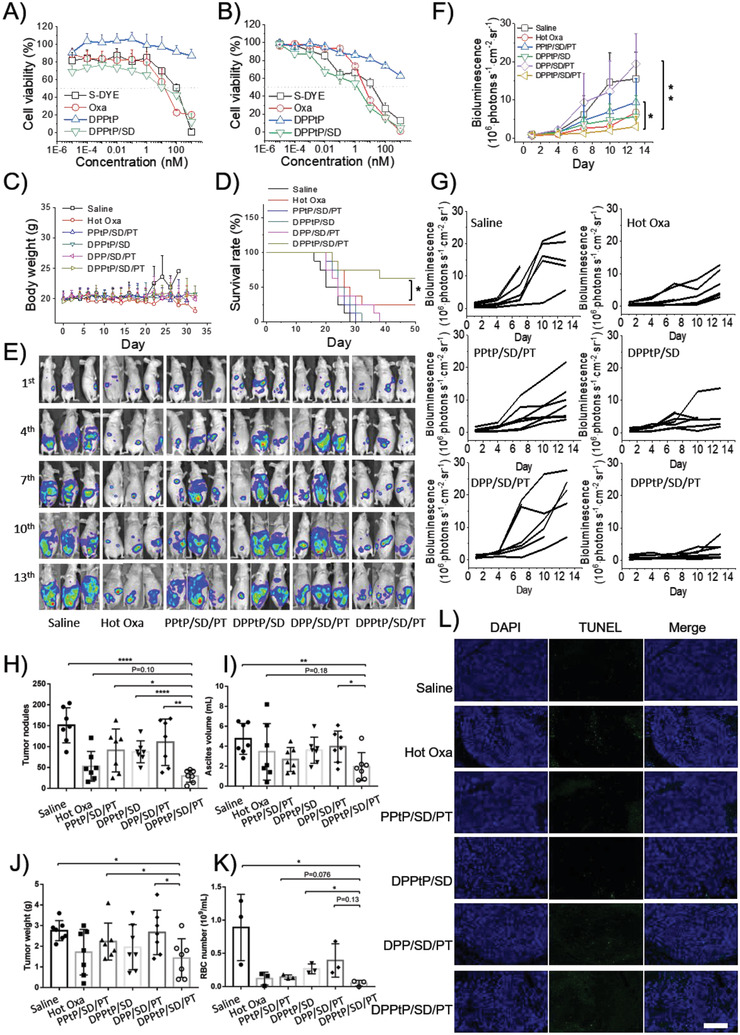
The antitumor efficacy evaluation. The cell viability of HCT‐116 cells treated with different formulations for 24 h at A) 37 or B) 43 °C by the CCK‐8 technique; C) the body weight change and D) the survival rate of the orthotopic tumor‐bearing mice during the therapy circle (*n* = 8); E) representative mice with bioluminescence indicating the tumor procession; F) summative and G) independent semiquantitative results of the mice with bioluminescence measured by IVIS during the therapy circle (*n* = 6); H) tumor nodules number, I) ascites volume, J) excised tumor weight (*n* = 7), and K) RBC number (*n* = 3) in ascites of the orthotopic tumor‐bearing mice during the therapy circle; and L) employing TUNEL to detect the in‐situ tumor cell apoptosis after the therapy circle, where the scale bar = 100 µm. (*p < 0.05, **p < 0.01, ***p < 0.001, or ****p < 0.0001).

Motivated by these above results, the antitumor efficacy was then evaluated. We first evaluated the cytotoxicity of HCT‐116 cells treated with different formulations for 24 h at 37 (**Figure** **7**A) or 43 °C (Figure 7B). The IC_50_ values for S‐DYE, Oxa, and DPPtP/SD are 103.3, 16.8, and 12.5 nm (37 °C)/14.0, 4.0 and 1.4 nm (37 °C), respectively. DPPtP/SD not merely inherited, but also enhanced the cytotoxicity from Oxa, possibly with the assistance of concomitant SO_2_. Hyperthermia could further improve the cytotoxicity by 8.9 folds, further suggesting the hyperthermia‐enhanced efficacy.

The in vivo antitumor efficacy was conducted on two orthotopic CCPM mice models. On the HCT‐116 cell‐derived CCPM mice model (human‐ sourced), DPPtP/SD/PT exhibited the highest survival rate (Figure 7D) with limited body weight variation (Figure 7C). On the CT‐26‐luci cell‐derived CCPM mice model, DPPtP/SD/PT demonstrated the most favorable antitumor efficacy as well, including the tumor growth (Figure 7E–G), tumor nodules number (Figure 7H), ascites volume (Figure 7I), excised tumor weight (Figure 7J), and red blood cell (RBC) number in ascites (Figure 7K). TUNEL was further employed to detect the in‐situ tumor cell apoptosis after the therapy circle (Figure 7L), where DPPtP/SD/PT also represented the most favorable formulation and tactic.

Traditional chemotherapy for CCPM therapy is featured by high toxicity, poor targeting, and will always impair the immune system due to obvious adverse effects.^[^ ^45^ ^]^ Hot Oxa, mimicking clinical protocol, did and can suppress the tumor growth but with nonnegligible body weight loss. DPPtP/SD/PT exhibited the most satisfactory antitumor results and limited invasiveness (Figures S30–S32, Supporting Information), of which the improved effect derived from the synergistic contribution of hyperthermia‐enhanced chemotherapy, deep penetration, perfused O_2_, plus the leveraged GSH‐ROS imbalance in tumor cells.

### 2.9 Safety Evaluation

**Figure 8:**
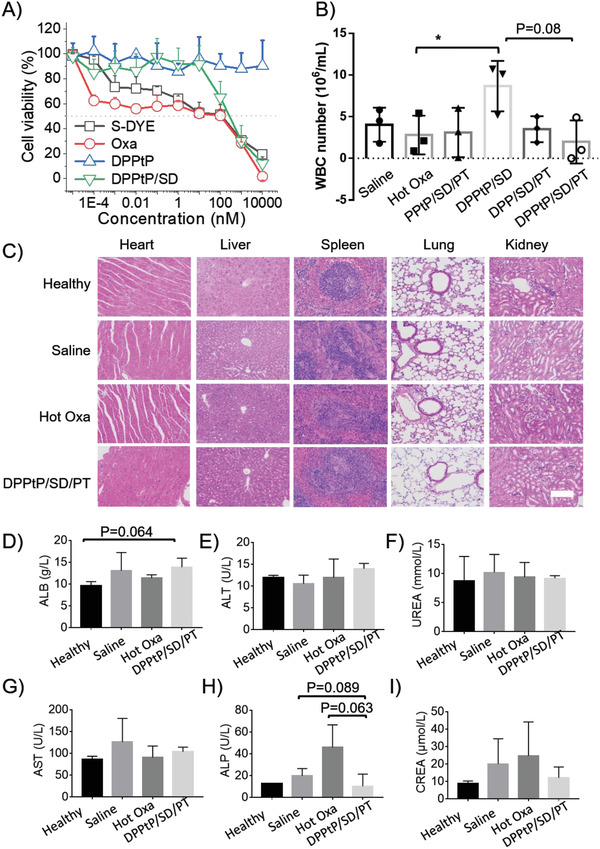
The biosafety evaluation. A) The cell viability of HEK‐293 cells treated with different formulations for 24 h at 37 °C by the CCK‐8 technique; B) the WBC number in ascites of the orthotopic tumor‐bearing mice during the therapy circle (*n* = 6); C) histochemistry analysis of heart, liver, spleen, lung, and kidney sections from different mice stained with hematoxylin and eosin (scale bar = 50 *μ*m); D) ALB, E) ALT, F) UREA, G) AST, H) ALP, and I) CREA levels in the blood from different mice (*n* = 3, *p < 0.05).

It is beneficial of simultaneously improving the therapeutic efficacy and systemic safety that the in vivo toxicity of the formulations was tested. In vitro cytotoxicity to HEK‐293 cells suggest that DPPtP/SD show decreased toxicity than parental Oxa (the IC_50_ values for S‐DYE, Oxa, and DPPtP/SD were measured to be 136.9, 95.1, and 299.2 nm (37 °C), respectively; **Figure** **8**A), possibly due to the uncomplete Oxa‐reconversion during the evaluation triggered by GSH, which is not so rich in somatic cells than tumor cells.

The white blood cell (WBC) numbers in ascites after the therapy circle was also assessed, and the DPPtP/SD/PT group show the lowest value (Figure 8B), indicating the reduced abdominal inflammation. Interestingly, only the DPPtP/SD group was detected with obvious inflammation, evidencing that the introduction with thermia treatment into the therapy protocol would inhibit the inflammation.

Though Pt‐based anticarcinoma drugs are participant in ≈70–80% clinical protocols, they are frequently hindered with strong physiological toxicity. Embedding Oxa into DPPtP/SD/PT can apparently reduce the side effect by the histochemistry analysis (Figure 8C). The hepatic and renal injury was also alleviated in the DPPtP/SD/PT group compared with hot Oxa treatment, by monitoring the clinicopathological parameters of ALB, ALT, UREA, AST, ALP, and CREA levels in the blood after the therapy circle (Figure 8D–I), suggesting favorable systemic toxicity for the proposed formulation.

## 3 Conclusion

HIPEC using Oxa is still displaying as the main weapon fighting against CCPM and recommended by clinical treatment guidelines worldwide, however, the strong systemic toxicity, intrinsic high invasiveness, and complicated modalities of HIPEC greatly limit its application. In this work, to reduce the side effect, we adopted a tumor‐targeting micelle to escort Oxa, which was expected to be liberated upon encountering a tumor microenvironment. To address the high invasiveness, we employed noninvasive photothermal means to generate the in‐situ hyperthermia for increasing local O_2_ level, deepening drug penetration, and enhancing the apoptosis. The developed “off‐to‐on” type photosensitizer could also help track the tumor niches scattered onto the peritonaeum by dual optical means (fluorescence and PA, “let there be light”^[^ ^46^ ^]^). Complicated modalities would thus be simplified by this proposed strategy, though upon meeting clinical translation in the future, minimally invasive laparoscopy should be further introduced into the whole system, to provide more accurate pertinency into enterocoelia, and to evade the contingent risk of insufficient penetration of NIR light into the tissues.

In this work, we proposed an alternative strategy for HIPEC in CCPM treatment by developing a versatile theranostics platform to provide spatial and temporal control on drug delivery and heat deposition. The versatile tumor‐targeting stimuli‐responsive platform, is a conceptually‐new type of smart theranostic platform for PMCC with capabilities of tumor‐tracking and photothermally enhanced chemotherapy.^[^ ^47^ ^]^ Hopefully, this system can be applied academically for “Quo Vadis” in the future, as well as substantially increase the clinical survival period of PMCC patients.

## 4 Experimental Section

### Materials

All starting chemicals were purchased from Sigma‐Aldrich (Shanghai, China). NMR reagents were from J&K Scientific Ltd. (Beijing, China). Oxaliplatin was purchased from Meilun Biotechnology Co. Ltd. (Dalian, China). All other compounds were from Sinopharm Chemical Reagent (Shanghai, China) and used as received. All polymers were from Jenkem (Beijing, China). DMEM and fetal bovine serum (FBS) were from Thermo Fisher Scientific Inc. (Waltham, USA). All detection kits were acquired from KeyGEN BioTECH (Nanjing, China).

### Equipment

^1^H NMR was obtained on a Varian Oxford NMR spectrometer (Palo Alto, CA, USA), while ^13^C NMR was obtained on a Bruker NMR Ascend 600 spectrometer (Billerica, Massachusetts, USA). MS‐ESI was determined on Agilent LCMSD (Santa Clara, CA, USA). The size and *ζ*‐potential of the aggregates in solution were recorded by DLS, Zetasizer Nano‐ZS, Malvern, U.K. The micromorphology was observed TEM (JEM‐2010, JEOL, Tokyo, Japan). The reconversion and release kinetics was measured by HPLC (Agilent Technologies Inc., CA, USA), equipped with gradient flow control pump (1260 Quat Pump‐G1311B), autosampler (1260 ALS‐G1329B), diode array detector (1260 DAD‐G4212B), fluorescence detector (FLD‐G1321A), and column oven (1290 TCC‐G1316C). A 250–4.6 column (RP‐C18 [5 µm], Agilent Technologies Inc., CA, USA). The cellular uptake was measured and photographed using a fluorescence microscope (Leica, Wetzlar, Germany) and flow cytometry (MoFloAstrios EQ, Beckman, CA, USA). A small animal imaging system (Image Visualization and Infrared Spectroscopy, IVIS) was undertaken on a Molecular Biology Workstations (Caliper, MA, USA). Pt determination was measured on ICP‐AES (iCAP PRO, Thermo Fisher Scientific, MA, USA). Molecular weight distribution of polymers was measured on MALDI‐TOF MS (AB Sciex, MA, USA). The fluorescence recovery on a cell level was recorded by a plate reader (BioTek, VT, USA). FLECT from TriFoil Imaging (www.TriFoilImaging.com) stands for Fluorescence Emission Computed Tomography. The FLECT system was a high‐resolution deep tissue in vivo optical tomography imaging platform that enables researchers to realize the full potential of in vivo fluorescence imaging for small animals. The FLECT system allows researchers to capture the 3D fluorescence signal in vivo in small laboratory animals with millimeter resolution.

### Chemical Synthesis

A retro‐synthesis was carried on before each synthesis. DYE, S‐DYE, and all polymers were synthesized according to the designed routes as illustrated in Figure S3, Supporting Information. The steps and full characterizations were provided in Figures S1–S16, Supporting Information.

### CMC Determination

The classic pyrene fluorescence probe method was used to determine the CMC value. Upon the formation of a micellar structure at a certain concentration, pyrene would be enveloped into the micellar core to result the fluorescence intensity (*λ*_ex/em_ = 334/480 nm) variation. The pyrene stock solution (10 µL, 2 × 10^–4^ mol L^−1^ in acetone) was added into vials, and the solvent was allowed to evaporate in the air at dark. Then the PEG‐Pt‐PLGA solutions with different concentrations were added to the vials and stirred overnight at r.t. to allow a full pyrene encapsulation into the micelles. The fluorescence intensity comparison was undertaken and plotted as a function of PEG‐Pt‐PLGA concentrations to give the CMC value at the inflexion.

### Drug Formulation

The classic film‐dispersion and hydration‐sonication method was employed to prepare the micelle. Briefly, S‐DYE, DPPtP, and PPtP with appropriate equivalence were dissolved into methanol, and methanol was completely removed under vacuum to yield a film on the flask wall, to which distilled water was added to the flask under sonication for 1 h to yield the micellar solution. Before DLS and TEM characterizations, the micelle was filtered through a 0.22 µm membrane. The stability test was carried on under 4 °C, and the loading capacity and encapsulation rate was evaluated by HPLC.

### Oxa and DYE Reconversion

Fluorescence spectrometer was employed to assess the recovered DYE from S‐DYE in presence of GSH at 37 °C in PBS 7.4/ACN (1/1, v/v). ICP‐AES was applied in determining the Pt distribution in vivo, while the molecular weight of the polymers distribution was measured by MALDI‐TOF MS. Vc was used to decompose the micelle and to trigger Oxa‐release at 37 °C. To determine Oxa‐release by HPLC, a UV detector at 250 nm was used. The mobile phase was methanol/H_2_O (1/9, v/v) for 15 min with a 1.0 mL min^−1^ flow rate. Under these conditions, Oxa exhibited a retention time at 6.2 min. The lower limit of the standard curve was 5 ng, and the injection volume was 20 µL for each submission. All solvents for Oxa and its derivate were chloride anion‐free.

### Cellular Uptake

HCT‐116 cells were cultured with DMEM added with 10% fetal bovine serum (FBS) and 1% v/v cocktail of antibiotic. 2 × 10^4^ HCT‐116 cells per well were seeded in 24 well plates (Corning‐Coaster, Tokyo, Japan), cultured at 37 °C for 72 h, and monitored under a microscope until reaching an 70% confluency with similar morphology.

Before each test, the medium was replaced with *D*‐glucose‐free DMEM medium for 12 h to achieve a balance. The cells were then added with different formulations and maintained for 30 min before a rinse with *D*‐glucose‐free Hank's. The cells were digested with trypsin (+EDTA), centrifuged, and washed twice with *D*‐glucose‐free Hank's. The quantitative results were obtained from a flow cytometry (BD Biosciences, Bedford, MA, USA) with 650 nm excitation, where 10^4^ cellular events were collected for each analysis, and the cells without treatment were used as the blank control.

### Biodistribution In Vivo

Biodistribution in vivo was studied on the subcutaneous (i.v.) and orthotopic tumor‐bearing nude mice. The mice were observed by IVIS at different time points. The ex vivo images of excised main organs were perfused with saline, and isolated from the tumor‐bearing mice after desired duration post administration, rinsed with saline, and observed under IVIS 12 h. The semi‐quantification was undertaken by measuring the fluorescence intensity of the range of interest (ROI) to illustrate the drug distribution. Photoacoustic imaging was undertaken on Vevo LAZR (Visual Sonics, Toronto, Canada) installed with a YAG laser with optical parametric oscillator. The measuring distance ranged from 1.5 to 3 cm.

As to the tissue observation, the mice were anaesthetized, perfused with saline/4% paraformaldehyde and sacrificed. The tumors were excised, fixed in 4% paraformaldehyde for 12 h, frozen in the OCT embedding medium (Sakura, Torrance, CA, USA) at −80 °C, sliced with 20‐µm thickness on a cryotome Cryostat (Leica, CM 1900, Wetzlar, Germany), stained with DAPI, and visualized by a fluorescence microscope (Leica, Wetzlar, Germany).

### Evaluation on the Tumor Spheroids

HCT‐116 tumor spheroids were prepared using the hanging‐drop technique. Briefly, 25 mL drops of the 0.24% methylcellulose‐containing culture medium solution suspended with 10 000 cells were pipetted onto the lid of round dishes, which were subsequently inverted over a dish filling with 10 mL Hank's to maintain the humidity of the environment. Hanging drop cultures were incubated at 37 °C with 5% CO_2_ for 4 days. The obtained 3D HCT‐116 tumor spheroids were harvested by pipetting 10 mL DMEM gently onto the lid and suspended the tumor spheroid into the mixture. The tumor spheroids were carefully transferred to a 24‐well plate and treated with different formulations for 2 h, and washed thrice with Hank's, fixed with 4% formaldehyde for 30 min, observed under confocal fluorescence microscope (Carl ZeissLSM710, Wetzlar, Germany) sliced in 20 µm.

### In Vitro Cytotoxicity

The in vitro cytotoxicity study was evaluated via CCK‐8 assay. The cells were seeded in a 96‐well plate at a density of 10^3^ cells per well and incubated until an 80% confluence with similar morphology. The cells were then supplemented with the tested formulations with different concentrations for 48 h. Then, the medium was removed and the cells were washed with Hank's before the test using a CCK‐8 kit, and the cells without treatment were used as control.

### In Vivo Antitumor Efficacy

All animal benefits and experiments were complied with Institutional Animal Care and Use Committee of Fudan University. Female balb/c nude mice of around 20‐g body weight were from Department of Experimental Animals of Fudan University and raised under ultra‐clean standard conditions. The subcutaneous xenograft tumor model was established by subcutaneous injection of 10^6^ HCT‐116 cells in 120 µL of Matrigel (5 mg mL^−1^, BD Biosciences, Bedford, MA, USA) in Hank's into the back of nude mice. The orthotopic tumor‐bearing mice model from HCT‐116 cells was established by i.p. injection with 10^6^ HCT‐116 cells in 120 µL of Matrigel in Hank's into the nude mice. The orthotopic tumor‐bearing mice model from CT‐26 cells was established by direct i.p. injection with 10^4^ CT‐26‐luci cells in Hank's into the nude mice. All mice were observed and the conditions were recorded every day post the tumor establishment. At the required time, the mice were randomly divided into groups for the antitumor efficacy evaluation.

The antitumor therapy was realized by i.p. injecting the grouped mice with different formulations (an equal dose of 5 mg Pt/kg body weight) via tail vein for four times every 2 days. After 12 h post each treatment, a local abdomen NIR irradiation (0.3 W cm^−2^) was launched for 30 min for the groups with irradiation.

The bioluminescence signal was used to reflect the tumor progression for the CT‐26‐luci‐derived models. The body weight and bioluminescence signal were recorded every other day. The tumor nodules number, ascites volume, excised tumor weight, and RBC number in ascites of the orthotopic tumor‐bearing mice after the therapy circle were recorded after euthanasia. The euthanasia was undertaken once the mice were beyond the animal benefit guidelines. The tumors were carefully excised, collected and rinsed by PBS 7.4 for further photographs. TUNEL assay was performed with 5‐µm frozen tumor slices detected with a DNA fragmentation detection kit according to the regular protocols provided by the producer and observed by fluorescent microscope (Leica, Wetzlar, Germany).

### Safety Evaluation

After the therapy circle, the excised main organs (heart, liver, spleen, lung, and kidney) from the sacrificed mice were sliced and stained with hematoxylin and eosin, which were then photographed under the inverted fluorescent microscope (Leica, Wetzlar, Germany).

### Statistical Analysis and Software

Origin 2018 and GraphPad Prism 7.00 were employed in analyzing and presenting the data. Statistical comparisons were carried on by one‐way ANOVA, with significance *p* < 0.05. The chemical structures were drawn on ChemBio3D Ultra 12.0. To better learn the theoretical “off‐to‐on” mechanism, the computational calculations based on the density functional theory at a B3LYP/6‐31G(d) level were performed by Gaussian 16 package. The structures of DYE and S‐DYE were first optimized, and the density differential density Δ*ρ* was analyzed. From Figures 1B and 1C, obvious redistribution of the electron density can be found for DYE and S‐DYE.

## Conflict of Interest

The authors declare no conflict of interest.

## Supporting information

Supporting Information

## Data Availability

The data that support the findings of this study are available from the corresponding author upon reasonable request.
