# Supplementary material for: A Versatile Theranostic Platform for Colorectal Cancer Peritoneal Metastases: Real‐Time Tumor‐Tracking and Photothermal‐Enhanced Chemotherapy
Source: Adv Sci (Weinh). 2021 Aug 16;8(20):2102256. doi: 10.1002/advs.202102256 (PMC8529449; doi:10.1002/advs.202102256)
Supplement: Supplementary file 1 — Supporting Information [file ADVS-8-2102256-s001.pdf]

© 2021 Wiley-VCH GmbH

ADVANCED SCIENCE
Open Access

Supporting Information

for *Adv. Sci.*, DOI: 10.1002/advs.202102256

A Versatile Theranostic Platform for Colorectal Cancer Peritoneal Metastases: Real-Time Tumor-Tracking and Photothermal-Enhanced Chemotherapy

*Tao Sun, Guangping Zhang, Tingting Ning, Qinjun Chen, Yongchao Chu, Yifan Luo, Haoyu You, Boyu Su, Chao Li, Qin Guo, Chen Jiang**

Supporting Information

A Versatile Theranostic Platform for Colorectal Cancer Peritoneal Metastases: Real-Time Tumor-Tracking and Photothermal-Enhanced Chemotherapy

*Tao Sun, Guangping Zhang, Tingting Ning, Qinjun Chen, Yongchao Chu, Yifan Luo, Haoyu You, Boyu Su, Chao Li, Qin Guo, Chen Jiang**

Contents

1. **Strategy comparison**
2. **Synthesis and characterizations**
3. **Theoretical calculation**
4. **Reconversion procedures**
5. **Tumor tracking**
6. **Photothermal property**
7. **Antitumor efficacy**
8. **References**

1. Strategy comparison

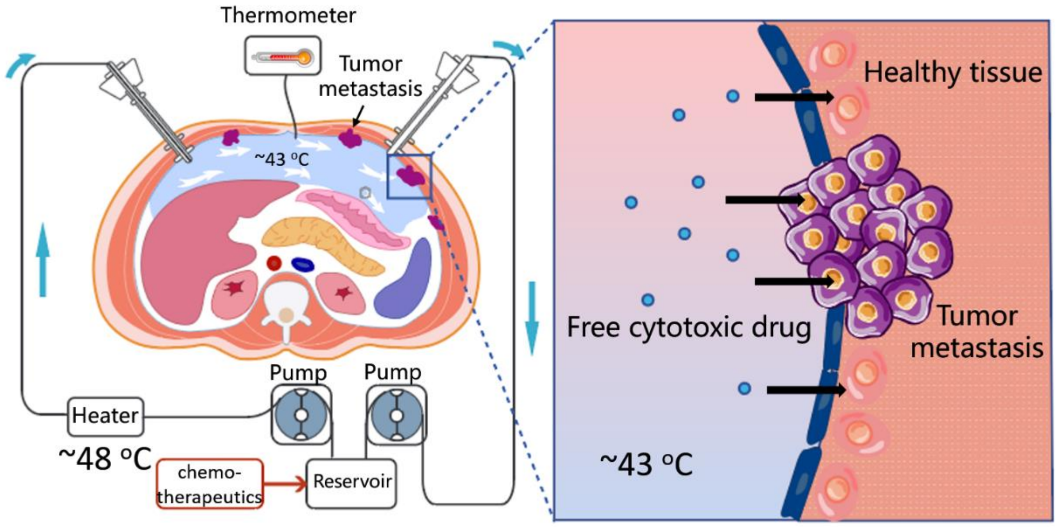

Scheme S1. Illustration of the conventional clinical therapeutic strategy, where complicated equipment should be required, and free cytotoxic drug tends to cause indiscriminate damage to tumors and healthy tissues upon being intraperitoneally injected.

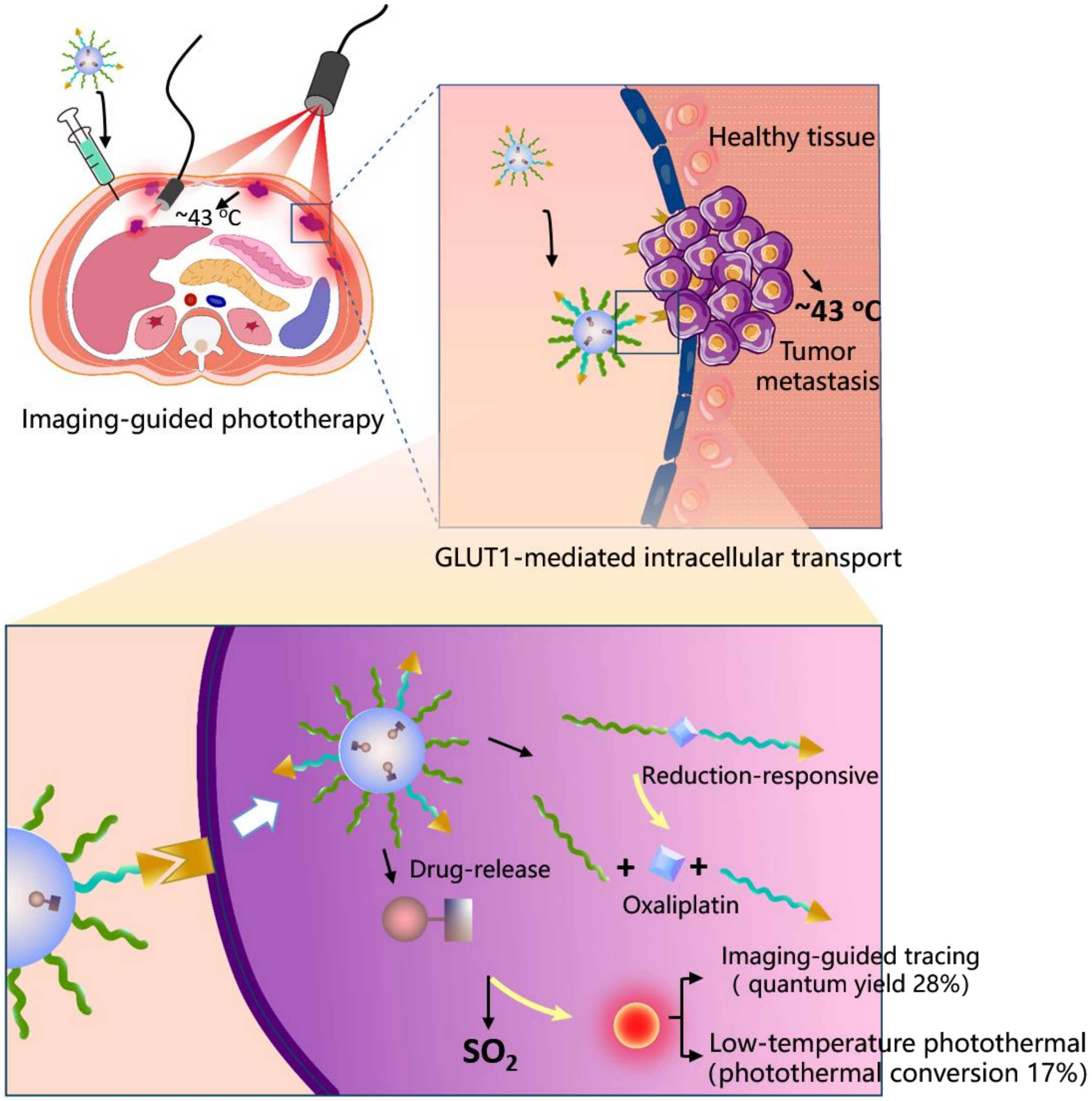

Scheme S2. The strategy proposed in this work, where imaging-guided low-temperature photothermal treatment was employed to assist the tumor-targeted chemotherapy.

2. Synthesis and characterizations

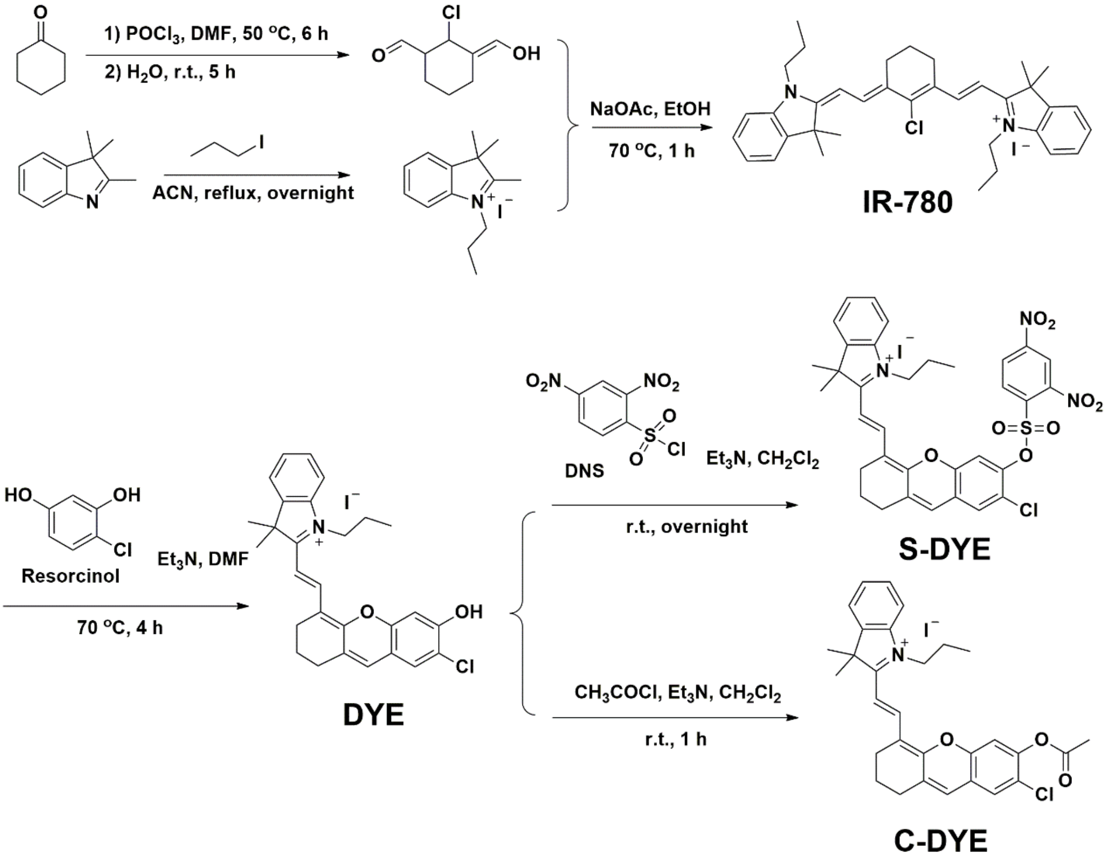

Scheme S3. Synthetic steps and structures of the photothermal sensitizer.

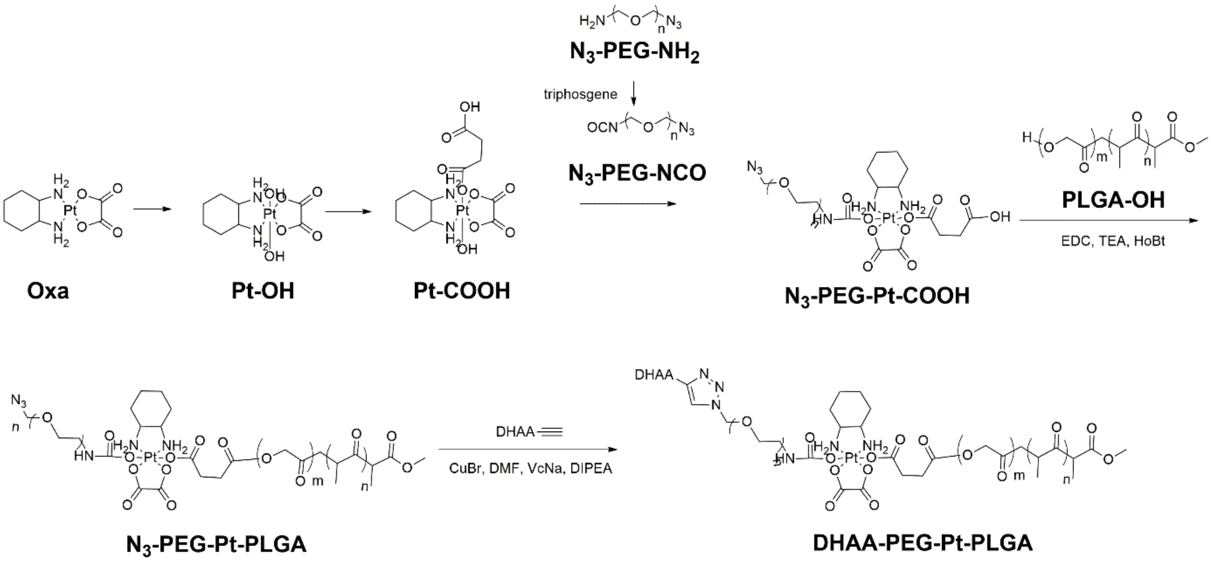

Scheme S4. Synthetic steps and structures of the Pt-embedded polymers.

Detailed steps as shown as below:

The molecule of DYE was synthesized and characterized according to our previous report[1].

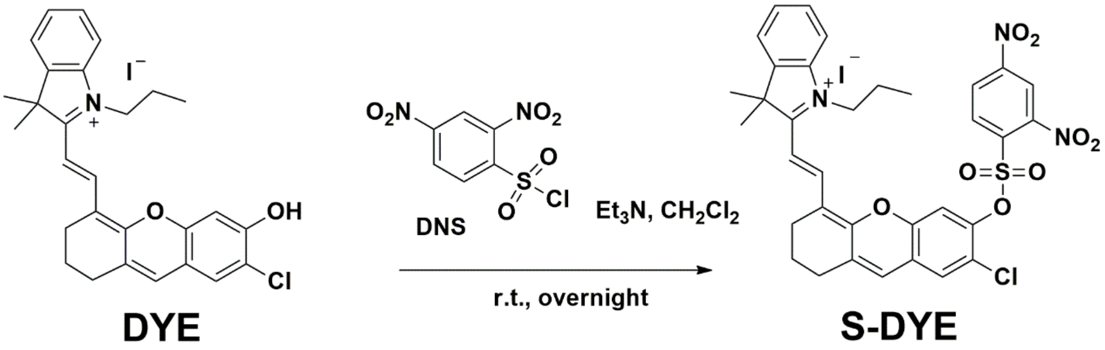

To be specific, to a solution of DYE (103.8 mg, 0.2 mmol, 1 eq) in anhydrous CH_2Cl_2 (10 mL) cooled to 0 ^oC, Et_3N (24.28 mg, 0.24 mmol, 1.2 eq) was added to the mixture in triple times in 10 min, during when, the temperature of the mixture was strictly maintained at 0 ^oC. Then, 2,4-dinitrobenzenesulfonyl chloride (DNS, 63.84 mg, 0.24 mmol, 1.2 eq) dissolved in anhydrous CH_2Cl_2 (10 mL) was slowly injected into the above solution in 1 h using a micro-injection pump. The obtained suspension was maintained at r.t./dark overnight under Ar. Then, CH_2Cl_2 was removed under vacuum to obtain the crude product, which was further purified by a silica gel column chromatography (DCM:mthanol=20:1, v:v) to afford S-DYE as a purple crystal with a yield of 42.78% and a UPLC-purity as 99.2% (Fig. S1). S-DYE was stored at -20 ^oC at dark.

R*f* = 0.4 (DCM:methanol=20:1, v:v).

^1H NMR (400 MHz, $CDCl_3$, δ, ppm): 8.699-8.368 (m, 3H, H-29~31), 8.317-8.269 (m, 1H, H-4), 7.565-7.483 (m, 1H, H-3), 7.335-7.265 (m, 3H, H-1, 2, 27), 6.990-6.843 (m, 3H, H-19, 26, 28), 2.813-2.693 (m, 4H, H-20, 21, 24, 25), 1.845 (s, 2H, H-7, 8), 1.586 (s, 5H, H-12~17), 1.065 (t, J= 6.8 Hz, 3H, H-9~11), 0.895-0.832 (m, 2H, H-22, 23).

^{13}C NMR (400 MHz, DMSO-d6, δ, ppm): 179.35, 174.34, 156.77, 151.57, 150.61, 147.95, 147.27, 145.20, 144.62, 144.56, 142.67, 141.05, 133.43, 132.28, 131.30, 130.59 (2C), 128.92, 128.15, 128.11, 127.50, 127.26, 125.48 (2C), 122.53, 121.86, 121.02, 118.16 (2C), 50.96 (2C), 28.85 (2C), 13.80.

MS-ESI Calc. for $C_{34}H_{31}ClN_3O_8S^+$ [**S-DYE**-I]$^+$ 676.2, Found, 676.2.

High resolution MS (HR-MS) Calc. for $C_{34}H_{31}ClN_3O_8S^+$ [**S-DYE**-I]$^+$ 676.1515, Found, 676.1519.

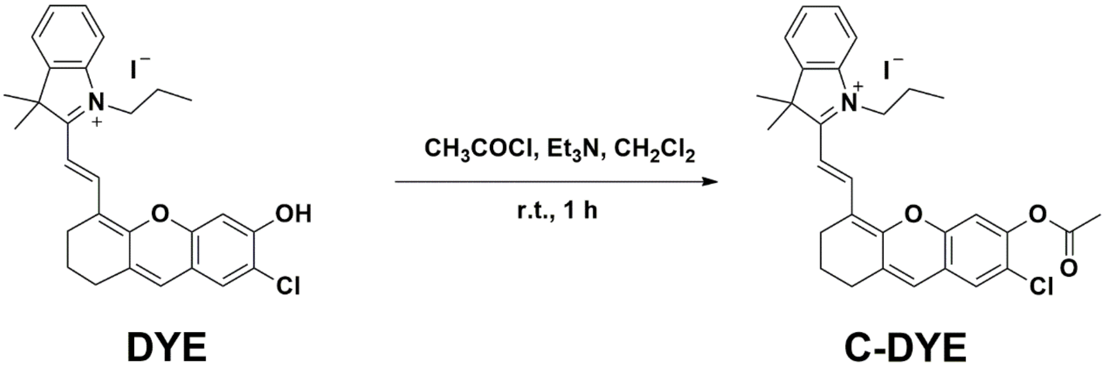

C-DYE was prepared as a control molecule, which cannot be "turned-on" in presence of GSH. The synthetic procedures were modified from a reported literature[2]. To a solution of DYE (82 mg, 0.16 mmol, 1 eq) in anhydrous CH_2Cl_2 (5 mL), Et_3N (45 μL, 33 mg, 0.32 mmol, 2 eq) was added. Then acetyl chloride (23 μL, 25 mg, 0.32 mmol, 2 eq) was added to the above solution at 25 ^oC and stirred for 1 h. The solvents was removed under vaccum and the crude product was purified by a silica gel column chromatography (DCM:mthanol=19:1, v:v) to afford **C-DYE** as a purple crystal with a yield of 49.04% and a UPLC-purity as 98.7% (Fig. S6). **C-DYE** was stored at -20 ^oC at dark.

R*f* = 0.6 (DCM:methanol=19:1, v:v).

^{1}H NMR (400 MHz, $CDCl_3$, δ, ppm): 7.957 (s, 1H, H-4), 7.736-7.643 (s, 2H, H-3, 27), 7.553 (d, J= 5.2 Hz, 2H, H-2), 7.477-7.381 (m, 3H, H-1, 26, 28), 7.218 (t, J=4.8 Hz, 1H, H-19), 6.372-6.254 (m, 1H, H-18), 4.139 (t, J=4.8 Hz, 2H, H-5, 6), 2.894 (s, 2H, H-20, 21), 2.734 (s, 2H, H-24, 25), 1.752 (t, J=7.2 Hz, 3H, H-29~31), 1.593-1.542 (m, 2H, H-22, 23), 1.091 (t, J=7.2 Hz, 3H, H-9~11), 0.940 (t, J=4.8 Hz, 2H, H-7, 8).

^{13}C NMR (400 MHz, DMSO-d6, δ, ppm): 171.70, 162.14 (2C), 160.92, 159.46, 141.99, 140.79, 140.06, 131.64, 130.46, 128.34, 124.75, 122.33, 120.65, 111.20, 100.38, 100.03, 87.86, 84.99, 69.61, 48.48, 36.62, 30.60, 28.86, 27.22 (2C), 26.98, 23.58, 20.16, 10.93.

MS-ESI Calc. for $C_{30}H_{31}ClNO_3^+$ [**S-DYE**-I]$^+$ 488.2, Found, 488.2.

High resolution MS (HR-MS) Calc. for $C_{30}H_{31}ClNO_3^+$ [**C-DYE**-I]$^+$ 488.1987, Found, 488.1976.

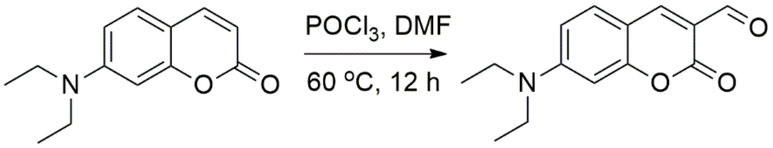

The synthesis of **SO_2-Probe** was following the reported steps[3]. Anhydrous *N*,*N*-dimethylformamide (DMF, 2 mL, 1.89 g, 26 mmol) was added dropwise to $POCl_3$ (2 mL, 3.29 g, 21 mmol) at 25 ^oC under an Ar atmosphere and further stirred for 30 min to give a

scarlet solution, which was then added with several portions of 7-diethylaminocoumarin (1.52 g, 7.0 mmol) dissolved in 10 mL DMF to yield a cardinal suspension. The mixture was maintained at 60 ^oC for 12 h and poured into ice water (150 mL, 0 ^oC). NaOH solution (20%, aq.) was added to adjust the pH to 10.0 to allow the formation of precipitation, which was filtered, washed with pure water, dried and recrystallized in absolute ethanol to give 7-diethylaminocoumarin-3-aldehyde (T.M., 1.08 g) in a 58% yield. The structure of **SO_2-Probe** was characterized by ^1H NMR, ^{13}C NMR and MS-ESI, which are in accordance with the reported reference[3].

R*f* = 0.5 (pure DCM).

^1H NMR (400 MHz, $CDCl_3$, δ, ppm): 10.130 (s, 1H, H-14), 8.259 (s, 1H, H-13), 7.422 (s, 1H. H-12), 6.64-6.62 (m, 1H. H-11), 6.481 (s, 1H. H-15), 3.472 (t, J=4.0 Hz, 4H, H-4, 5, 9, 10), 1.249 (d, J=4.0 Hz, 6H, H-1~3, 6~8).

^{13}C NMR (400 MHz, DMSO-d6, δ, ppm): 187.36, 161.29, 158.26, 152.83, 144.76, 131.90, 113.72, 109.54, 107.62, 96.54, 44.54 (2C), 11.93 (2C).

MS-ESI Calc. for $C_{14}H_{16}NO_3^+$ [**M+H**]$^+$ 246.1, Found, 246.0.

The compound of Pt-COOH was prepared following with our previous work[4].

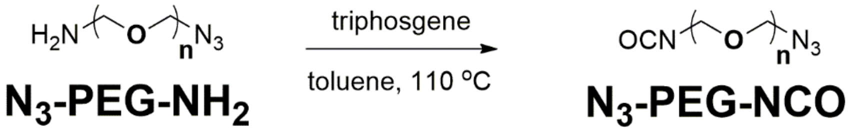

Anhydrous toluene was heated to reflux (110 ^oC) under Ar atmosphere and a solution of triphosgene (38.2 mg, 0.13 mmol, 1.3 eq) in anhydrous toluene was added. Then, a solution of N_3-PEG-NH_2 (5K, 530 mg, 1 mmol, 1 eq) in anhydrous toluene was added dropwise with a syringe in 1 h. The reaction mixture was stirred for 2 h at reflux. Then, the solvent was removed under reduced pressure to result N_3-PEG-NCO, which was directly used without purification.

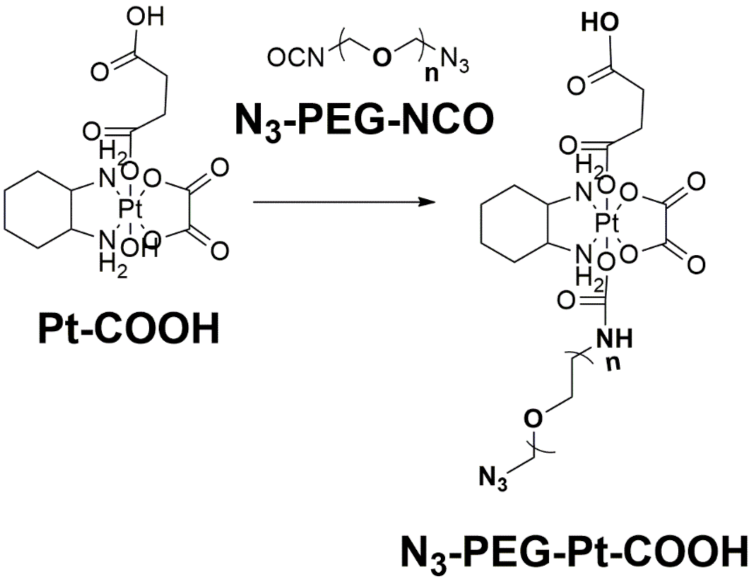

A solution of compound Pt-COOH (451 mg, 0.85 mmol) in dry DMF, plus catalytic amount of dibutyltin dilaurate (DBTL), was added to the above isocyanate residue dissolved in dry DMF. The reaction mixture was heated to 50 ^{o}C, stirred for 2 h under Ar atmosphere. Then, DMF was removed under vacuum. The residue was precipitated in Et_2O thrice to give N_3-PEG-Pt-COOH as a white solid.

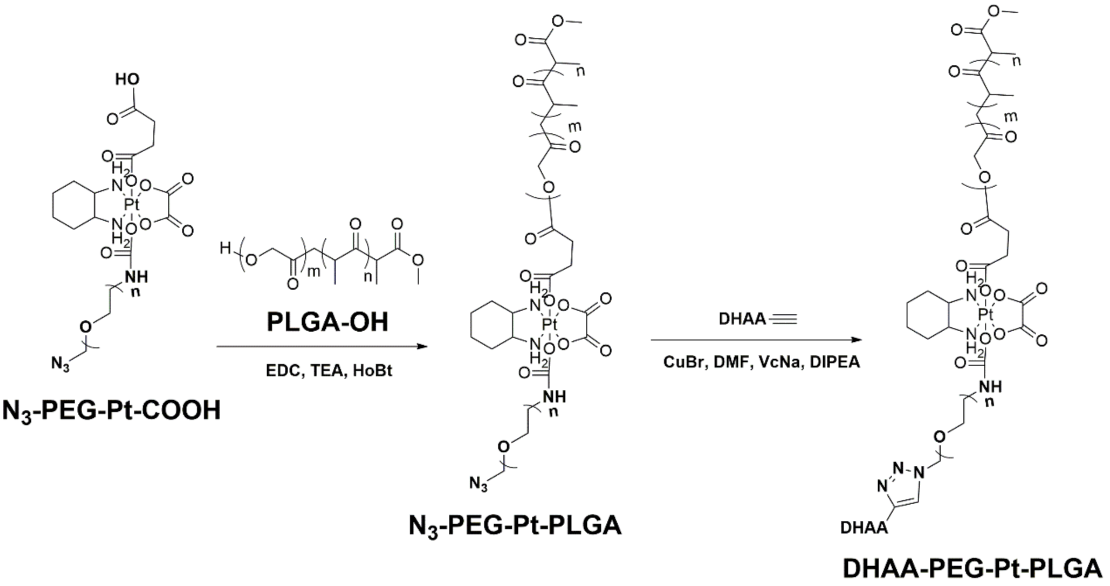

N_3-PEG-Pt-COOH (558 mg, 0.1 mmol, 1 eq), 1-(3-dimethylaminopropyl)-3-ethylcarbodiimide hydrochloride (EDC, 28.8 mg, 0.15 mmol, 1.5 eq), 1-hydroxybenzotriazole (HoBt, 20 mg, 0.15 mmol, 1,5 eq) and Et_3N (21 μL, 15.3 mg, 0.15 mmol, 1.5 eq) were dissolved in anhydrous DMF (5 mL) and stirred for 2 h, then added with PLGA-O H (5K, 500 mg, 0.1 mmol, 1 eq). The mixture was allowed to react at r.t. overnight. The suspension was dialyzed against DMF (1 L) for 48 h using a MWCO 8K. DMF was removed under vacuum,

and the residue solution was allowed to precipitate into Et_2O thrice to give N_3-PEG-Pt-PLGA as a white solid. The polymers were characterized with ^1H NMR and GPC.

PEG-Pt-PLGA was prepared following the same procedure, except for the replacement of N_3-PEG-NH_2 to PEG-NH_2.

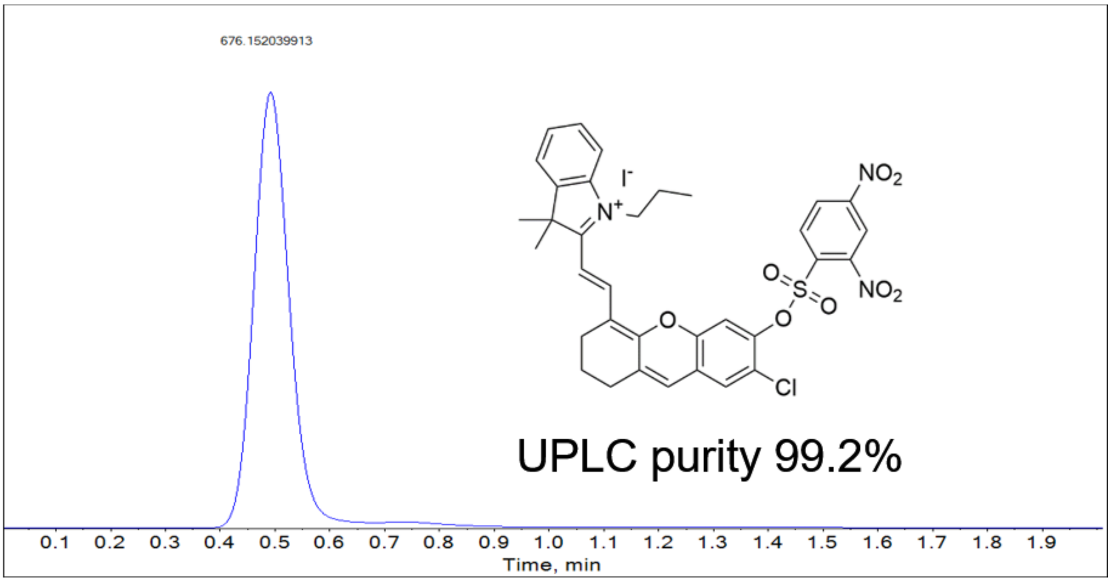

Fig. S1. The purity of **S-DYE** evaluated with UPLC.

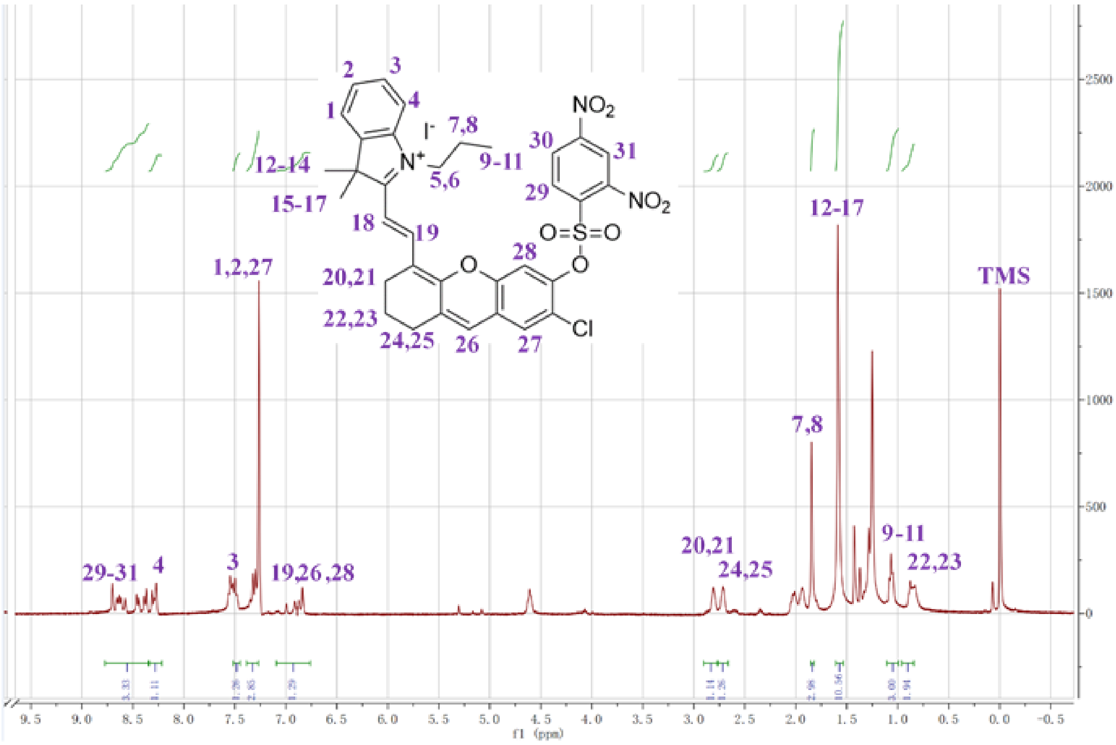

Fig. S2. ^1H NMR spectrum of **S-DYE** in DMSO-d6.

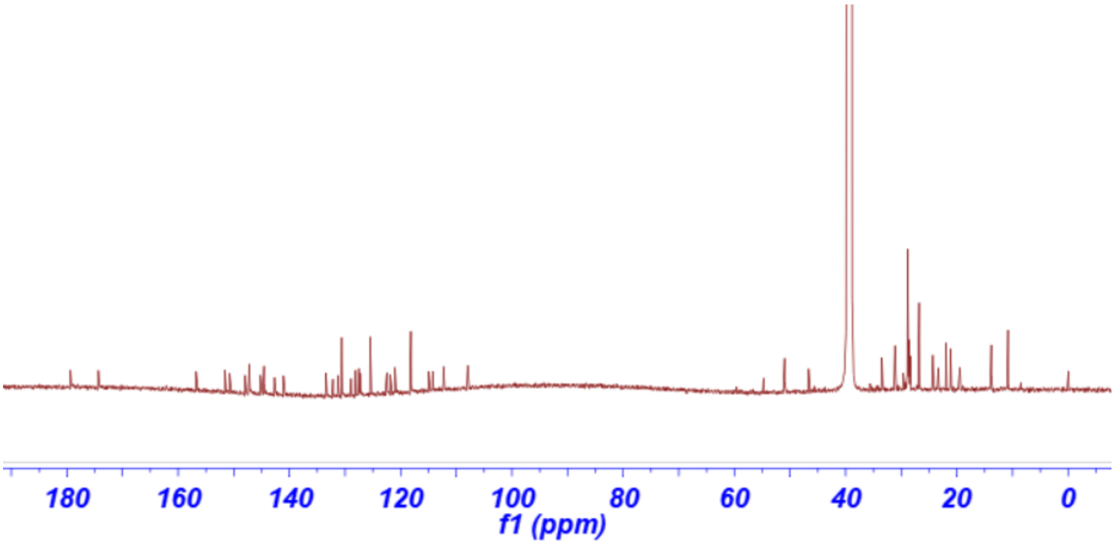

Fig. S3. ^{13}C NMR spectrum of **S-DYE** in DMSO-d6.

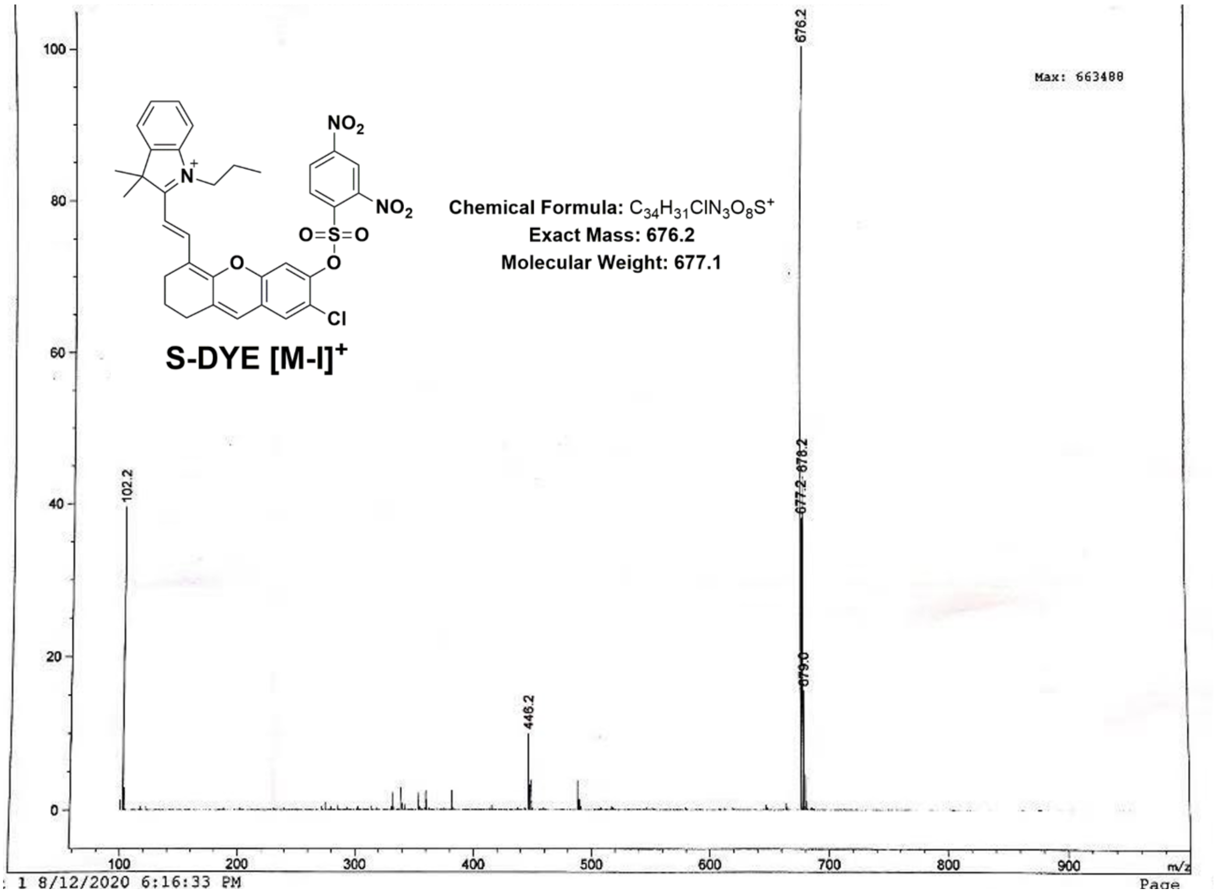

Fig. S4. The ESI-MS spectrum of **S-DYE**.

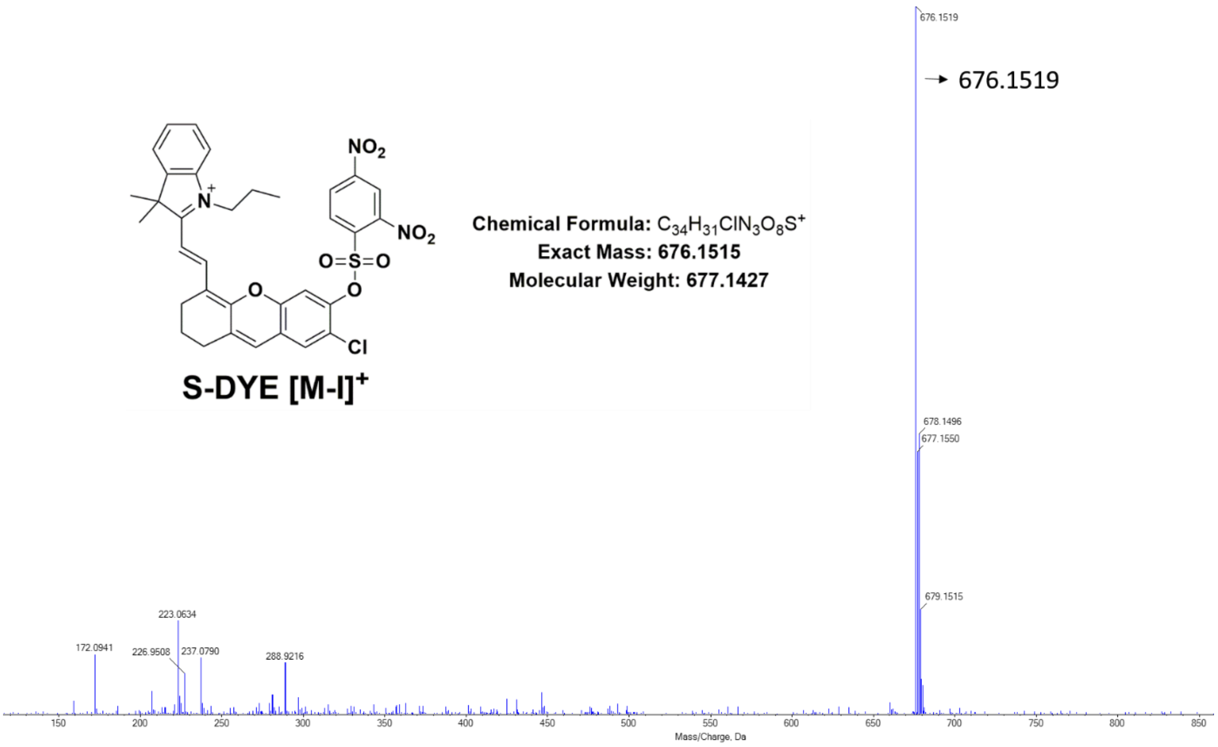

Fig. S5. The HR-MS spectrum of **S-DYE**.

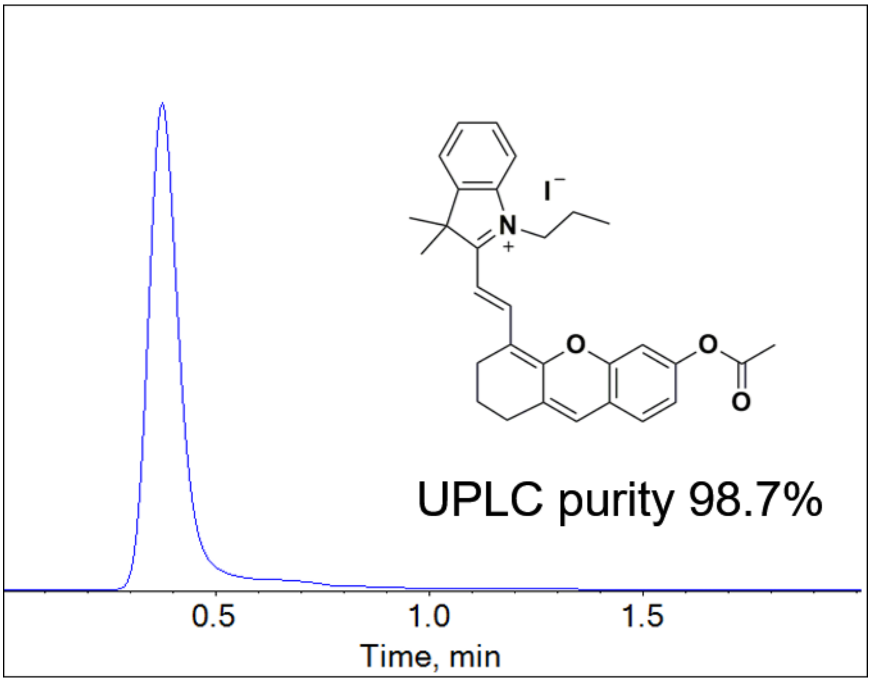

Fig. S6. The purity of **C-DYE** evaluated with UPLC.

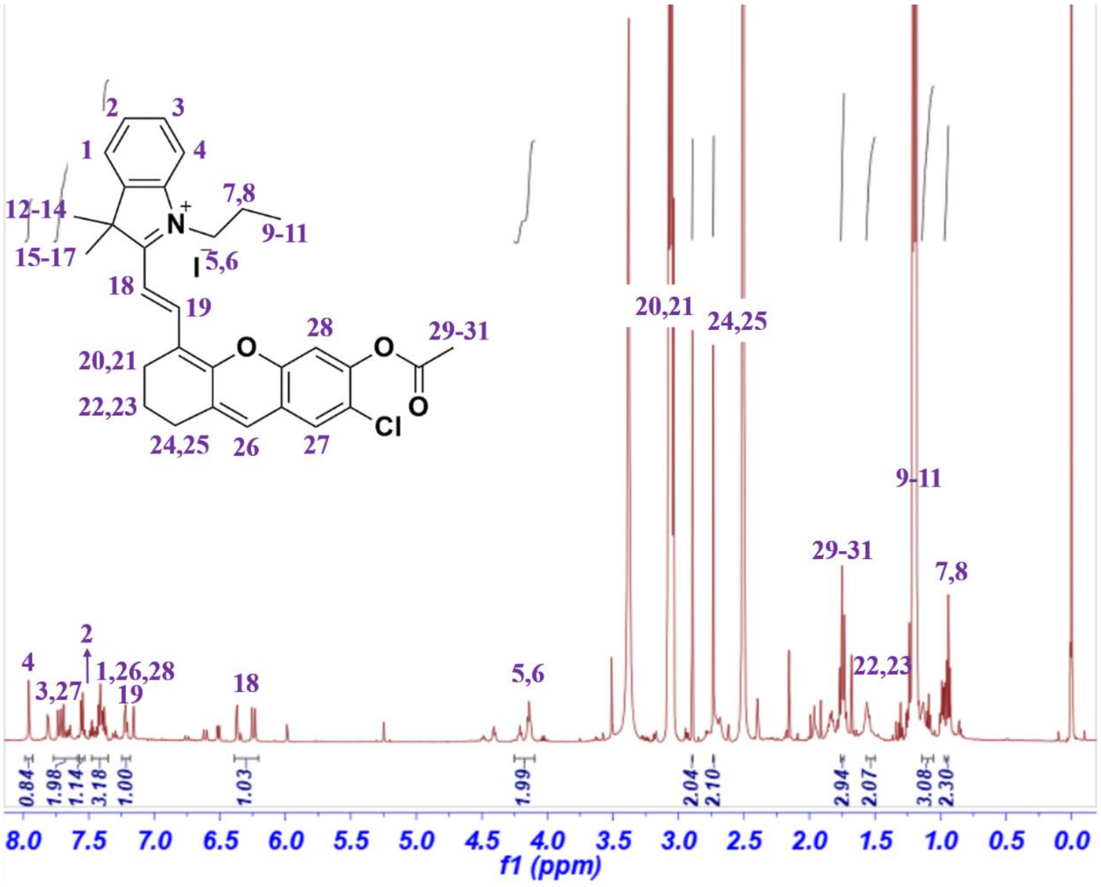

Fig. S7. ^{1}H NMR spectrum of **C-DYE** in DMSO-d6.

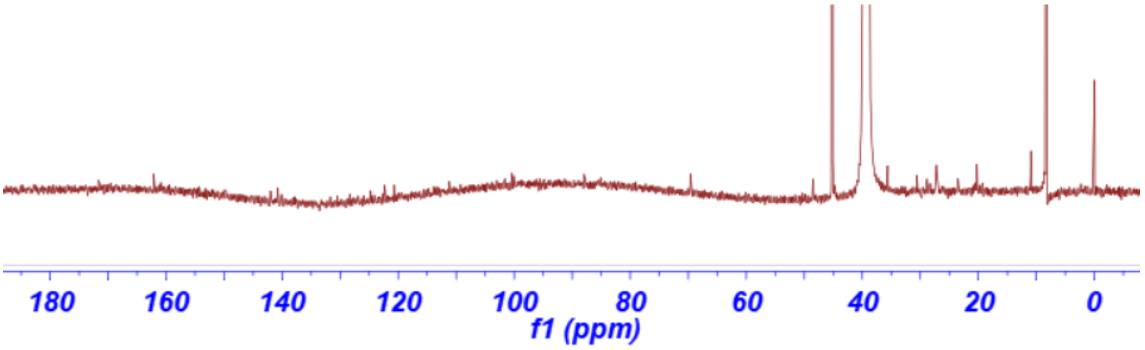

Fig. S8. ^{13}C NMR spectrum of **C-DYE** in DMSO-d6.

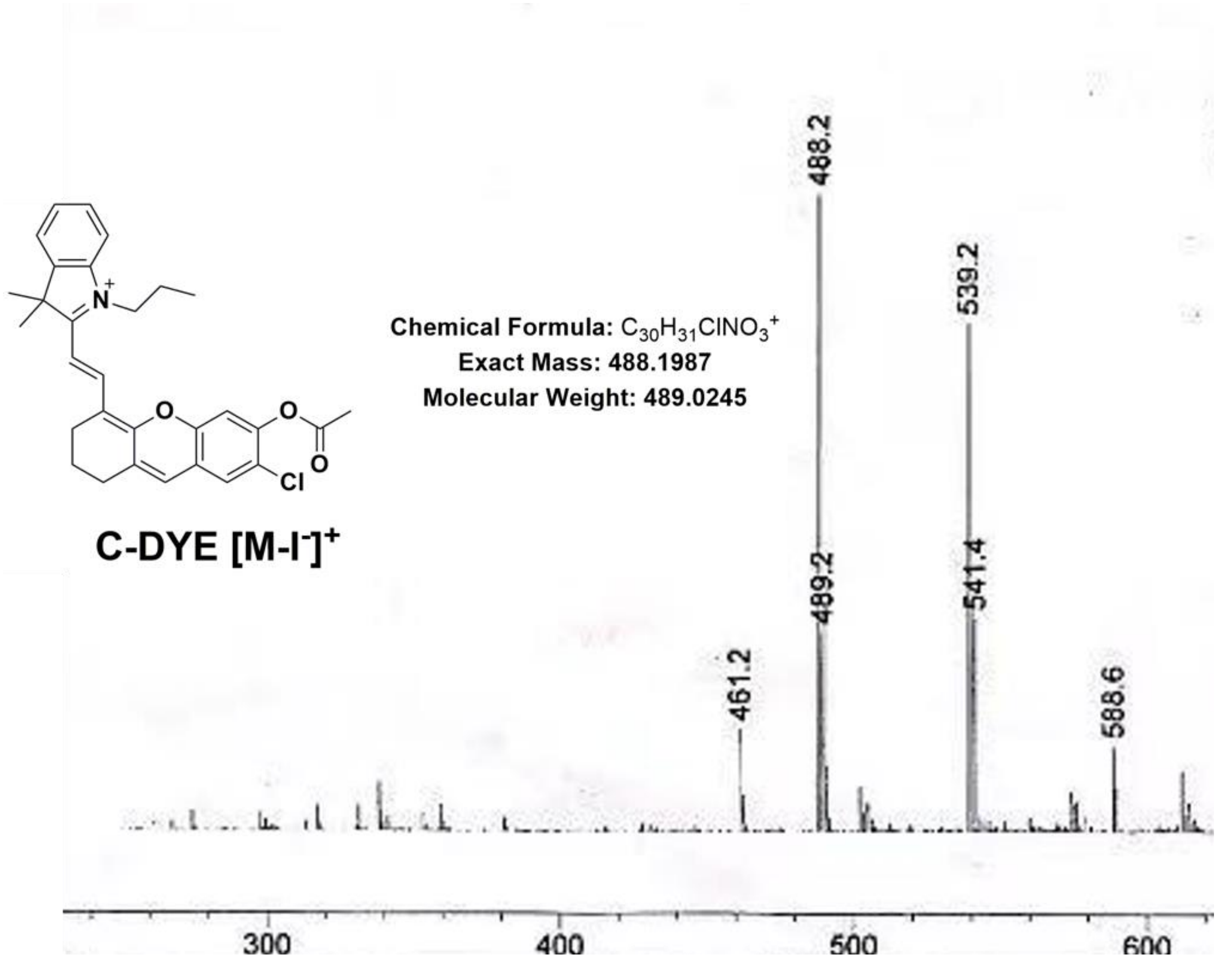

Fig. S9. The ESI-MS spectrum of **C-DYE** (MS of 539.2 is the interior label).

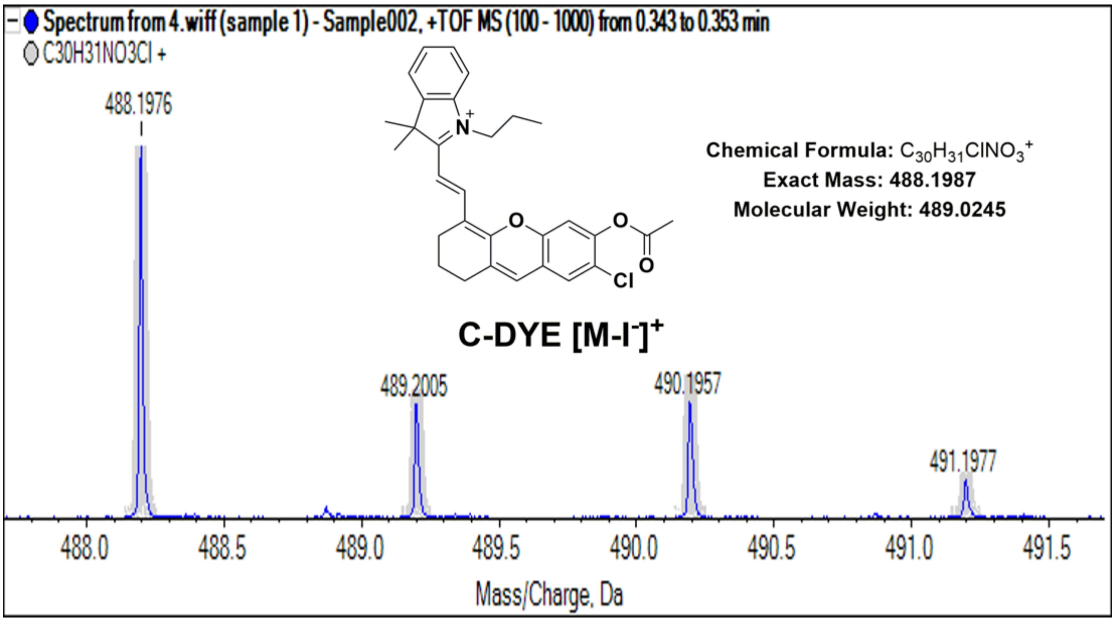

Fig. S10. The HR-MS spectrum of **C-DYE**.

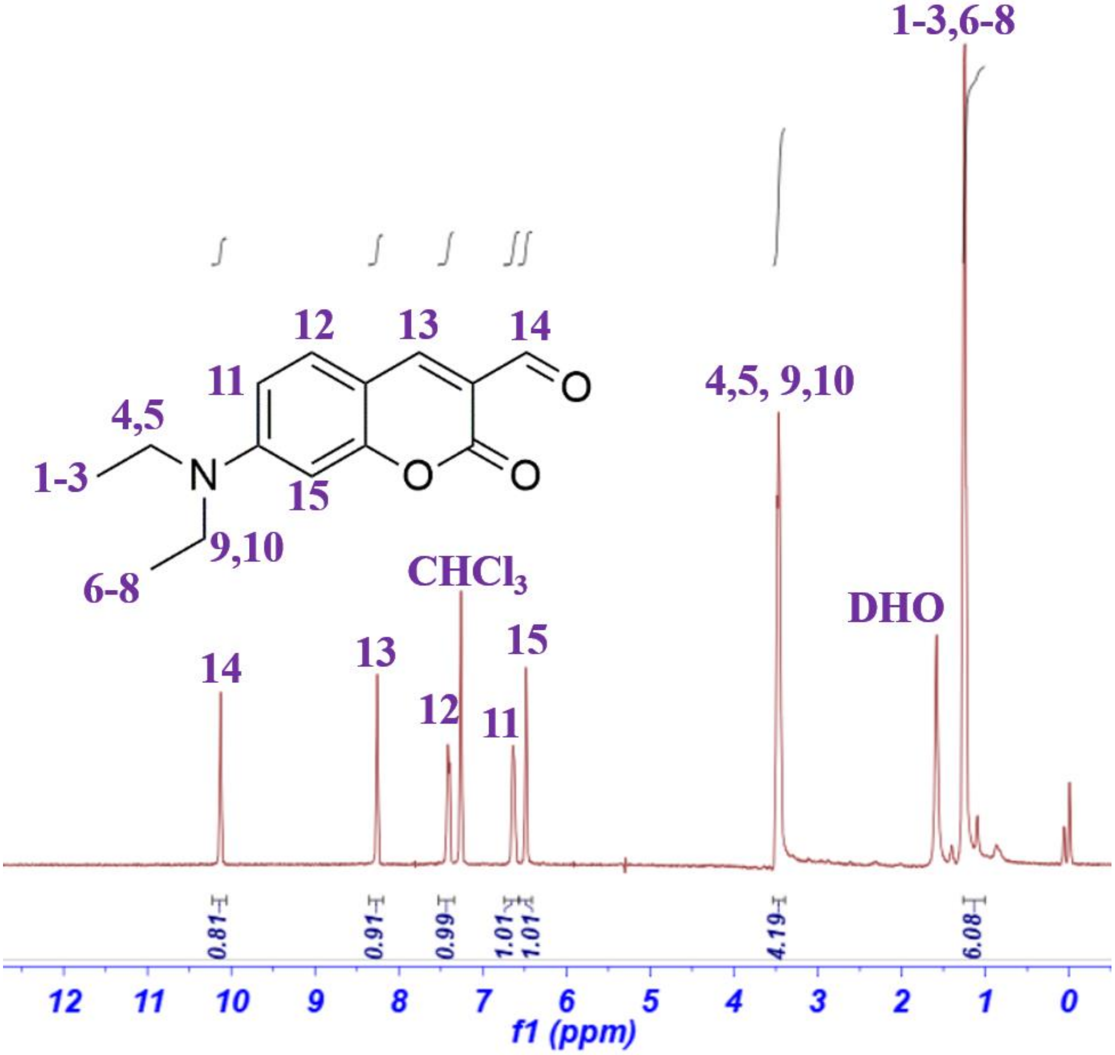

Fig. S11. ^{1}H NMR spectrum of **SO_2-Probe** in $CDCl_3$.

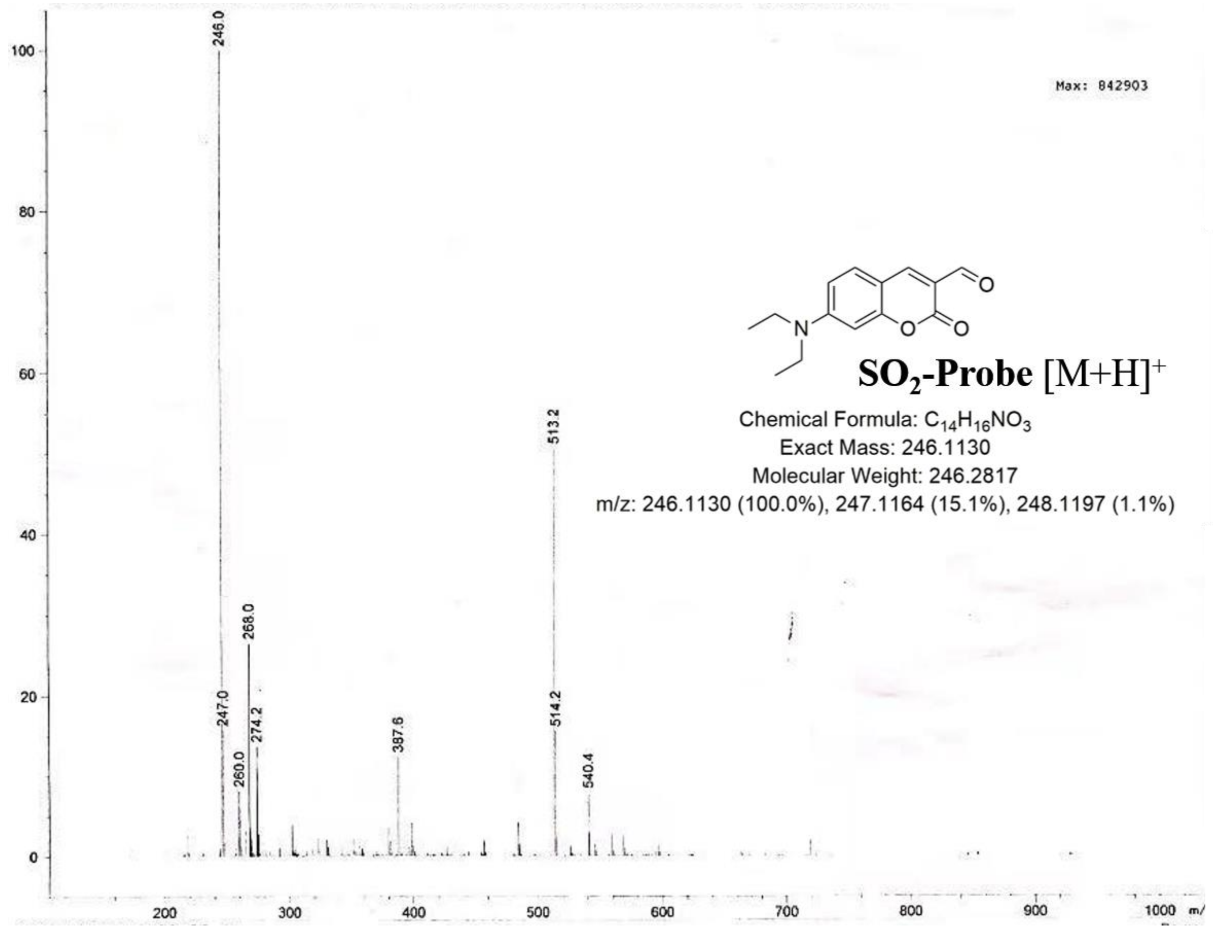

Fig. S12. The ESI-MS spectrum of **SO_2-Probe**.

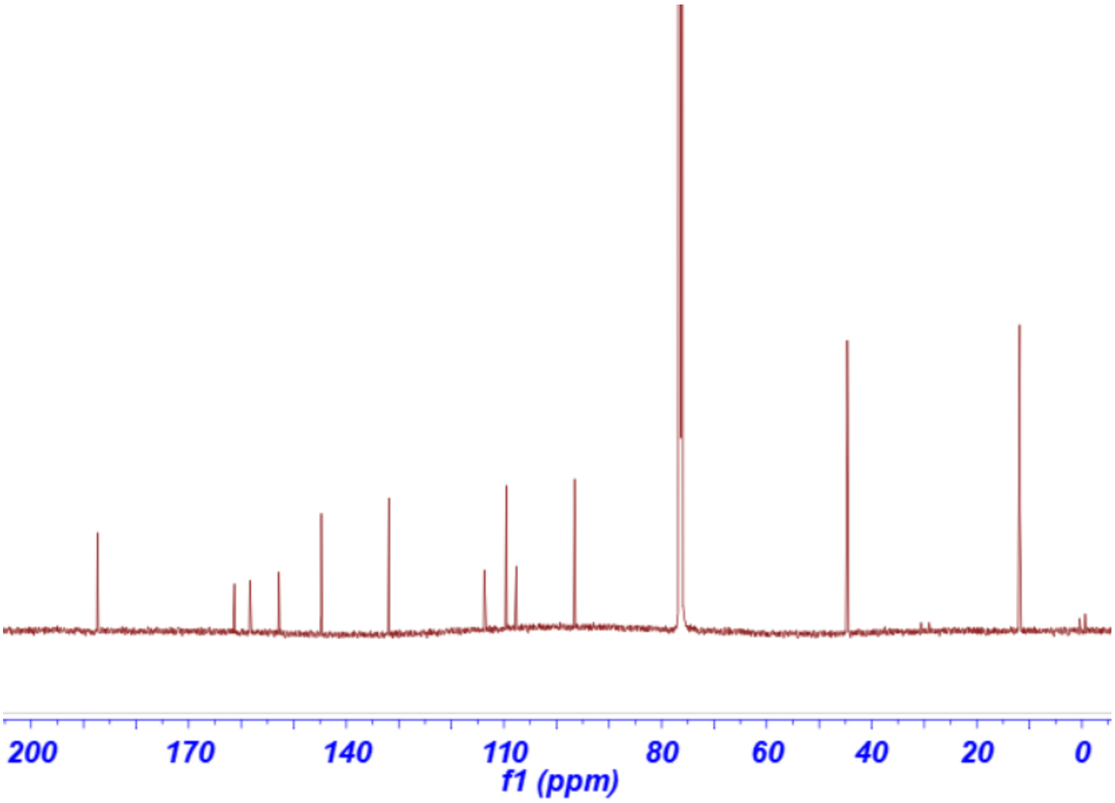

Fig. S13. ^{13}C NMR spectrum of **SO_2-Probe** in DMSO-d6.

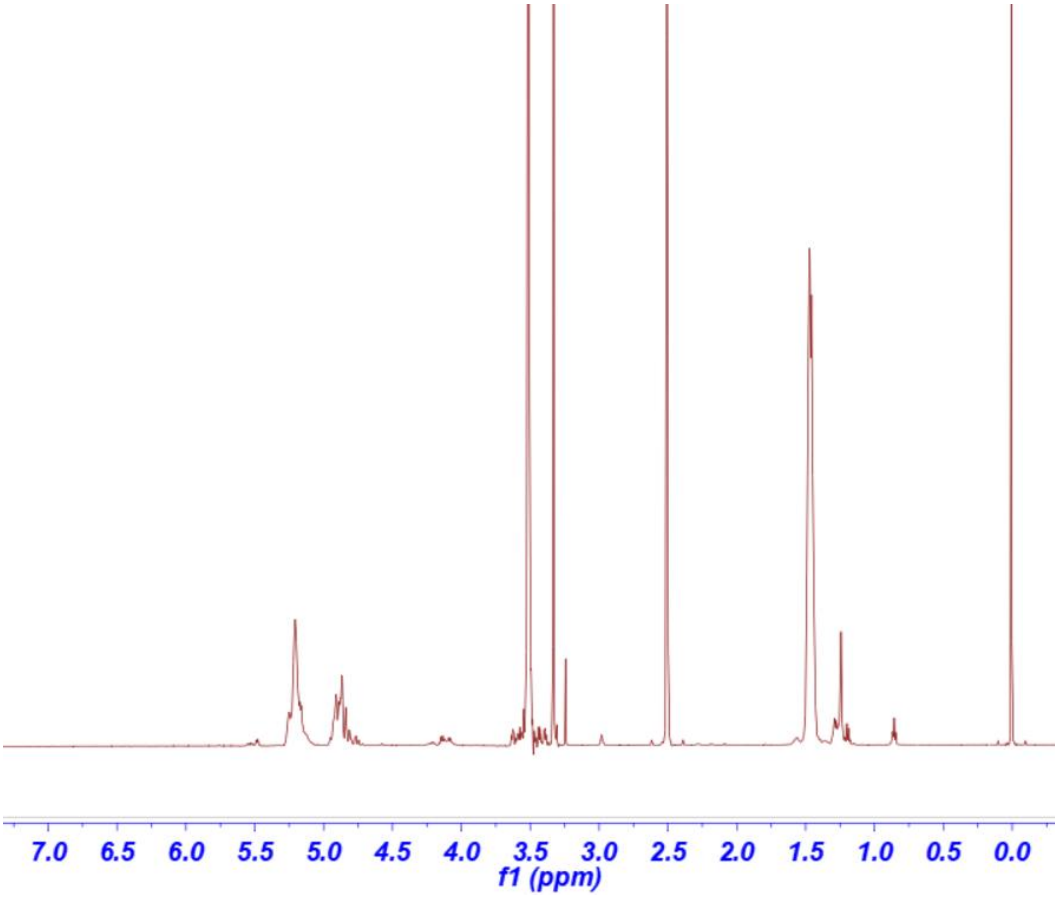

Fig. S14. ^{1}H NMR spectrum of **PEG-Pt-PLGA** in DMSO-d6.

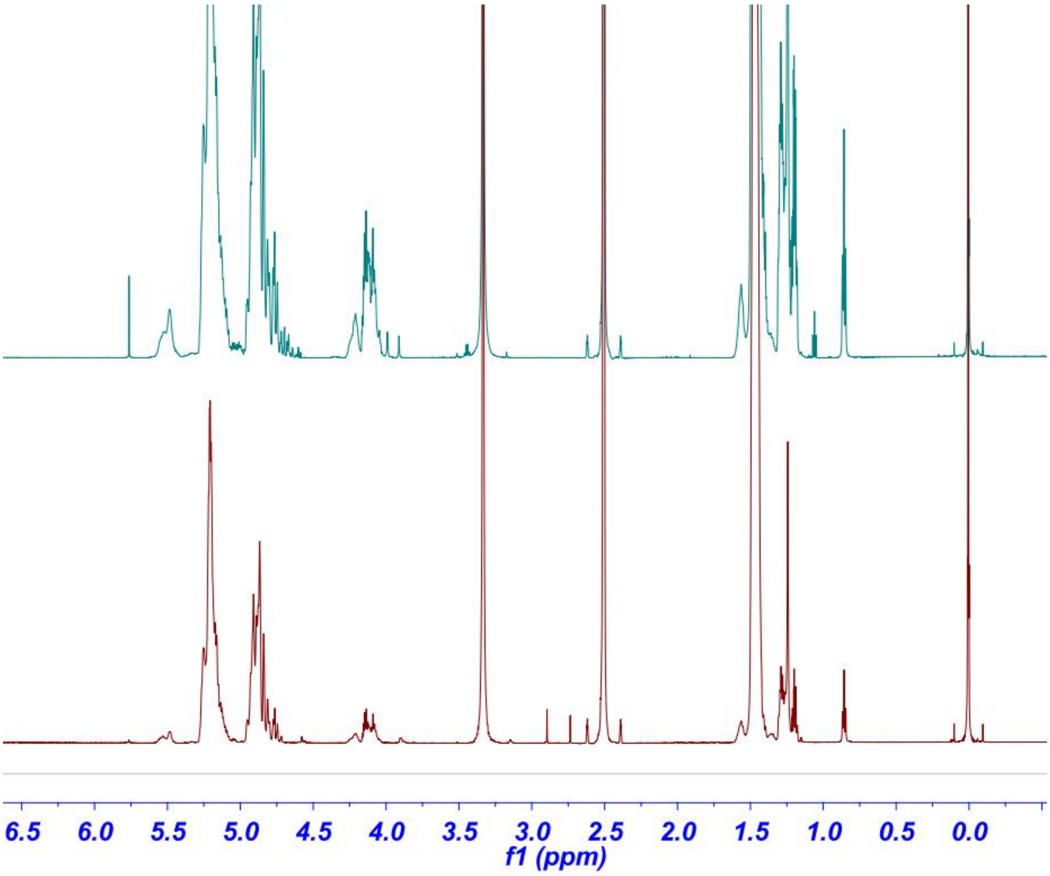

Fig. S15. ^{1}H NMR spectrum of **N_3-PEG-Pt-PLGA** (up, green) and **DHAA-PEG-Pt-PLGA** (down, red) in DMSO-d6.

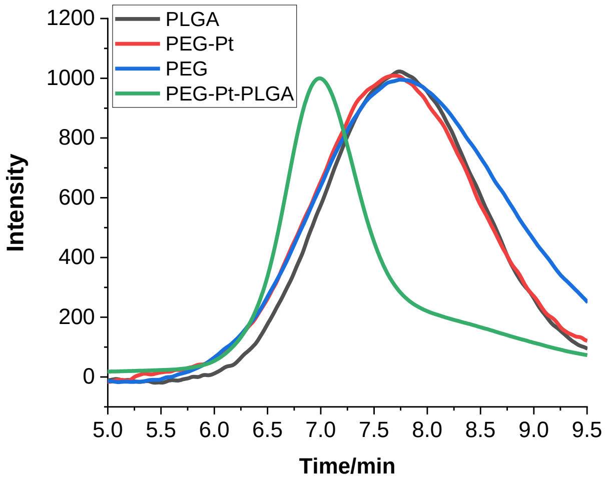

Fig. S16. GPC results of **PLGA**, **PEG-Pt**, **PEG** and **PEG-Pt-PLGA** using DMF as the flowing phase, where the retention time of **PEG-Pt-PLGA** is clear decreased to suggest the combination of the molecular weight from **PLGA** and **PEG**.

3. Theoretical calculation

	DYE	S-DYE
Wavelength found/nm	679/730	571/NA
Wavelength calculated/nm	535.6/662.2	532.5/**1463.7**

Table S1. The found and calculated wavelength of DYE and S-DYE, respectively. Different from traditional "off-to-on" probe, the absorbance of S-DYE doesn't vary much upon the modification, while the emission displays a clear red-shift to silent the probe based on the calculation results.

Calculated absorbance spectrometer of **DYE** in aqueous solution:

```
Excited State   1:      Singlet-A      2.3150 eV  535.56 nm  f=1.3419  <S**2>=0.000
Excited State   2:      Singlet-A      3.3015 eV  375.53 nm  f=0.0276  <S**2>=0.000
Excited State   3:      Singlet-A      3.5410 eV  350.14 nm  f=0.0038  <S**2>=0.000
Excited State   4:      Singlet-A      3.6357 eV  341.02 nm  f=0.0509  <S**2>=0.000
Excited State   5:      Singlet-A      3.6891 eV  336.08 nm  f=0.1999  <S**2>=0.000
Excited State   6:      Singlet-A      4.0505 eV  306.10 nm  f=0.1225  <S**2>=0.000
Excited State   7:      Singlet-A      4.2378 eV  292.57 nm  f=0.0518  <S**2>=0.000
Excited State   8:      Singlet-A      4.4586 eV  278.08 nm  f=0.0534  <S**2>=0.000
Excited State   9:      Singlet-A      4.7659 eV  260.15 nm  f=0.1264  <S**2>=0.000
Excited State  10:      Singlet-A      4.9202 eV  251.99 nm  f=0.0557  <S**2>=0.000
Excited State  11:      Singlet-A      5.0223 eV  246.87 nm  f=0.0007  <S**2>=0.000
Excited State  12:      Singlet-A      5.1421 eV  241.12 nm  f=0.0005  <S**2>=0.000
Excited State  13:      Singlet-A      5.1706 eV  239.79 nm  f=0.1761  <S**2>=0.000
Excited State  14:      Singlet-A      5.1886 eV  238.95 nm  f=0.0409  <S**2>=0.000
Excited State  15:      Singlet-A      5.2288 eV  237.12 nm  f=0.0134  <S**2>=0.000
Excited State  16:      Singlet-A      5.2844 eV  234.62 nm  f=0.0071  <S**2>=0.000
Excited State  17:      Singlet-A      5.3372 eV  232.30 nm  f=0.0189  <S**2>=0.000
Excited State  18:      Singlet-A      5.3917 eV  229.95 nm  f=0.0014  <S**2>=0.000
Excited State  19:      Singlet-A      5.4555 eV  227.27 nm  f=0.0362  <S**2>=0.000
Excited State  20:      Singlet-A      5.5606 eV  222.97 nm  f=0.0015  <S**2>=0.000
```

Calculated emission spectrometer of **DYE** in aqueous solution:

```
Excited State   1:      Singlet-A      1.8724 eV  662.16 nm  f=1.4708  <S**2>=0.000
Excited State   2:      Singlet-A      3.0644 eV  404.59 nm  f=0.1075  <S**2>=0.000
Excited State   3:      Singlet-A      3.4045 eV  364.17 nm  f=0.3631  <S**2>=0.000
Excited State   4:      Singlet-A      3.4089 eV  363.71 nm  f=0.0015  <S**2>=0.000
Excited State   5:      Singlet-A      3.4737 eV  356.93 nm  f=0.0230  <S**2>=0.000
Excited State   6:      Singlet-A      3.8767 eV  319.82 nm  f=0.1668  <S**2>=0.000
```

Table S2. The calculated absorbance and emission spectrometer of **DYE** in aqueous solution.

Calculated absorbance spectrometer of **S-DYE** in aqueous solution:

```
Excited State   1:      Singlet-A      1.8570 eV  667.65 nm  f=0.0009  <S**2>=0.000
Excited State   2:      Singlet-A      2.3282 eV  532.53 nm  f=1.0053  <S**2>=0.000
Excited State   3:      Singlet-A      2.3558 eV  526.30 nm  f=0.2668  <S**2>=0.000
Excited State   4:      Singlet-A      3.1065 eV  399.12 nm  f=0.0002  <S**2>=0.000
Excited State   5:      Singlet-A      3.3163 eV  373.86 nm  f=0.0520  <S**2>=0.000
Excited State   6:      Singlet-A      3.4182 eV  362.72 nm  f=0.0004  <S**2>=0.000
Excited State   7:      Singlet-A      3.4541 eV  358.94 nm  f=0.0107  <S**2>=0.000
Excited State   8:      Singlet-A      3.4650 eV  357.82 nm  f=0.2159  <S**2>=0.000
Excited State   9:      Singlet-A      3.5637 eV  347.91 nm  f=0.0561  <S**2>=0.000
Excited State  10:      Singlet-A      3.5975 eV  344.64 nm  f=0.0007  <S**2>=0.000
Excited State  11:      Singlet-A      3.6655 eV  338.24 nm  f=0.0282  <S**2>=0.000
Excited State  12:      Singlet-A      3.7223 eV  333.09 nm  f=0.0000  <S**2>=0.000
Excited State  13:      Singlet-A      3.7742 eV  328.50 nm  f=0.0141  <S**2>=0.000
Excited State  14:      Singlet-A      3.8863 eV  319.03 nm  f=0.0002  <S**2>=0.000
Excited State  15:      Singlet-A      3.9319 eV  315.33 nm  f=0.0000  <S**2>=0.000
Excited State  16:      Singlet-A      3.9385 eV  314.80 nm  f=0.0001  <S**2>=0.000
Excited State  17:      Singlet-A      4.0216 eV  308.29 nm  f=0.0005  <S**2>=0.000
Excited State  18:      Singlet-A      4.1778 eV  296.77 nm  f=0.0495  <S**2>=0.000
Excited State  19:      Singlet-A      4.2136 eV  294.25 nm  f=0.0251  <S**2>=0.000
Excited State  20:      Singlet-A      4.2197 eV  293.82 nm  f=0.1116  <S**2>=0.000
```

Calculated emission spectrometer of **S-DYE** in aqueous solution:

```
Excited State   1:      Singlet-A      0.8471 eV 1463.71 nm  f=0.0101  <S**2>=0.000
Excited State   2:      Singlet-A      1.7034 eV  727.87 nm  f=0.0024  <S**2>=0.000
Excited State   3:      Singlet-A      1.9959 eV  621.21 nm  f=1.4280  <S**2>=0.000
Excited State   4:      Singlet-A      2.1846 eV  567.54 nm  f=0.0123  <S**2>=0.000
Excited State   5:      Singlet-A      2.5617 eV  483.99 nm  f=0.0004  <S**2>=0.000
Excited State   6:      Singlet-A      2.6756 eV  463.39 nm  f=0.0002  <S**2>=0.000
```

Table S3. The calculated absorbance and emission spectrometer of **S-DYE** in aqueous solution.

4. Reconversion procedures

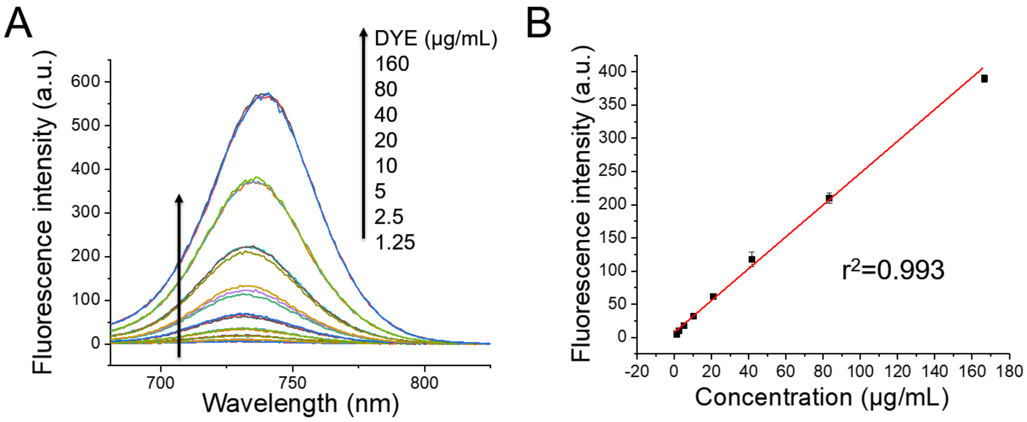

Fig. S17. A) The fluorescence intensity variation as the **DYE** concentration increases in mixed ACN:H_2O (1:1, v:v) (n=3); B) the standard curve of DYE in mixed ACN:H_2O (1:1, v:v) (n=3).

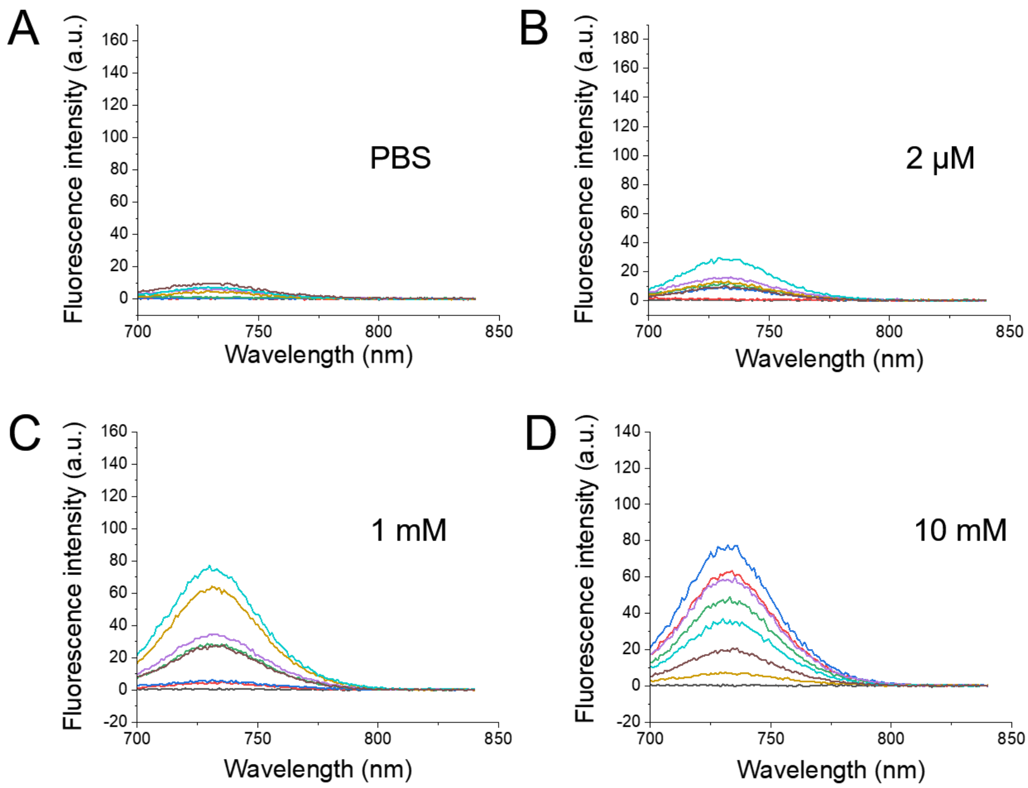

Fig. S18. A) The fluorescence intensity variation of **S-DYE** in mixed ACN:H_2O (1:1, v:v) in presence of GSH with different concentrations A) 0; B) 2 μM; C) 1 mM and D) 10 mM.

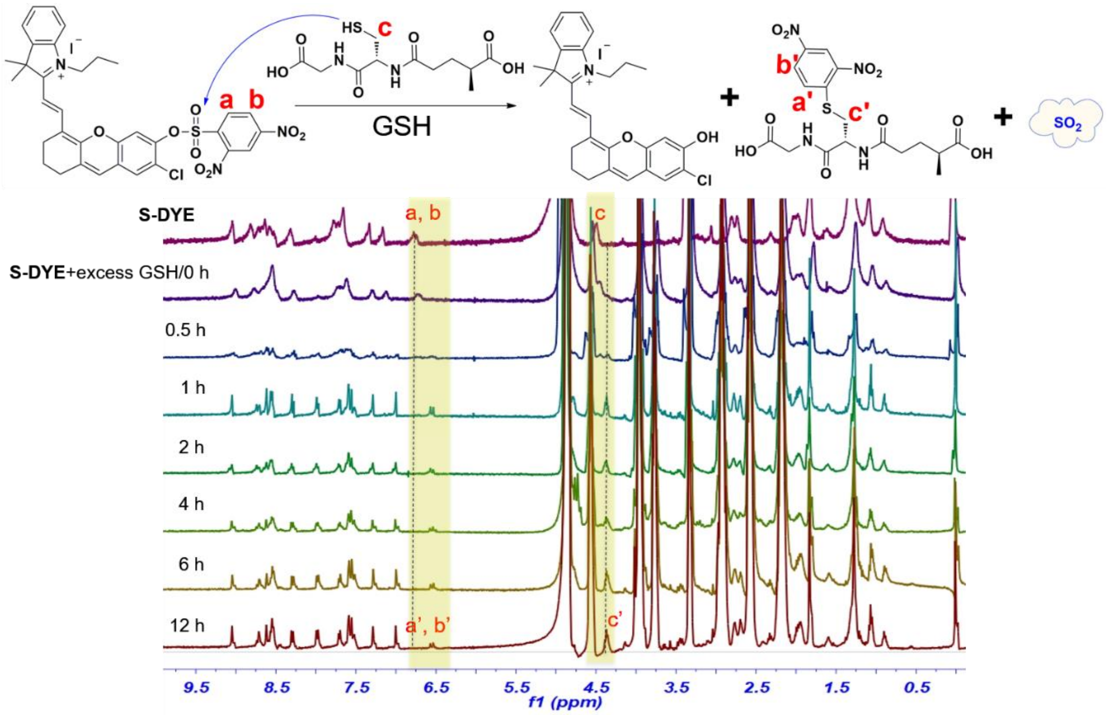

Fig. S19. The ^{1}H NMR variation over time of **S-DYE** in mixed MeOD:D_2O (1:1, v:v, 37 ^oC) in presence of excess GSH. Based on the above results, it is clear that thiol group of GSH can attack the sulphate on **S-DYE** and replace the 2,4-DNS moiety to liberate free **DYE** and SO_2.

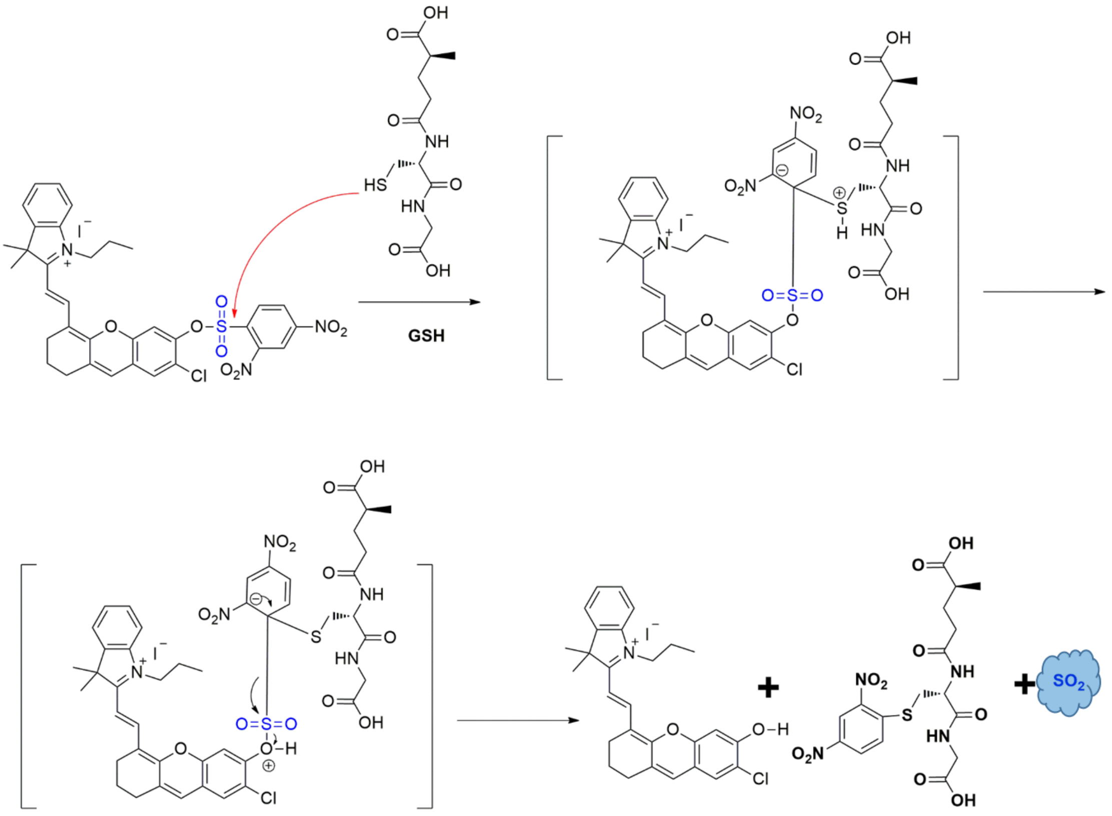

Fig. S20. The detailed SO_2-release mechanism initiated by GSH to **S-DYE**.

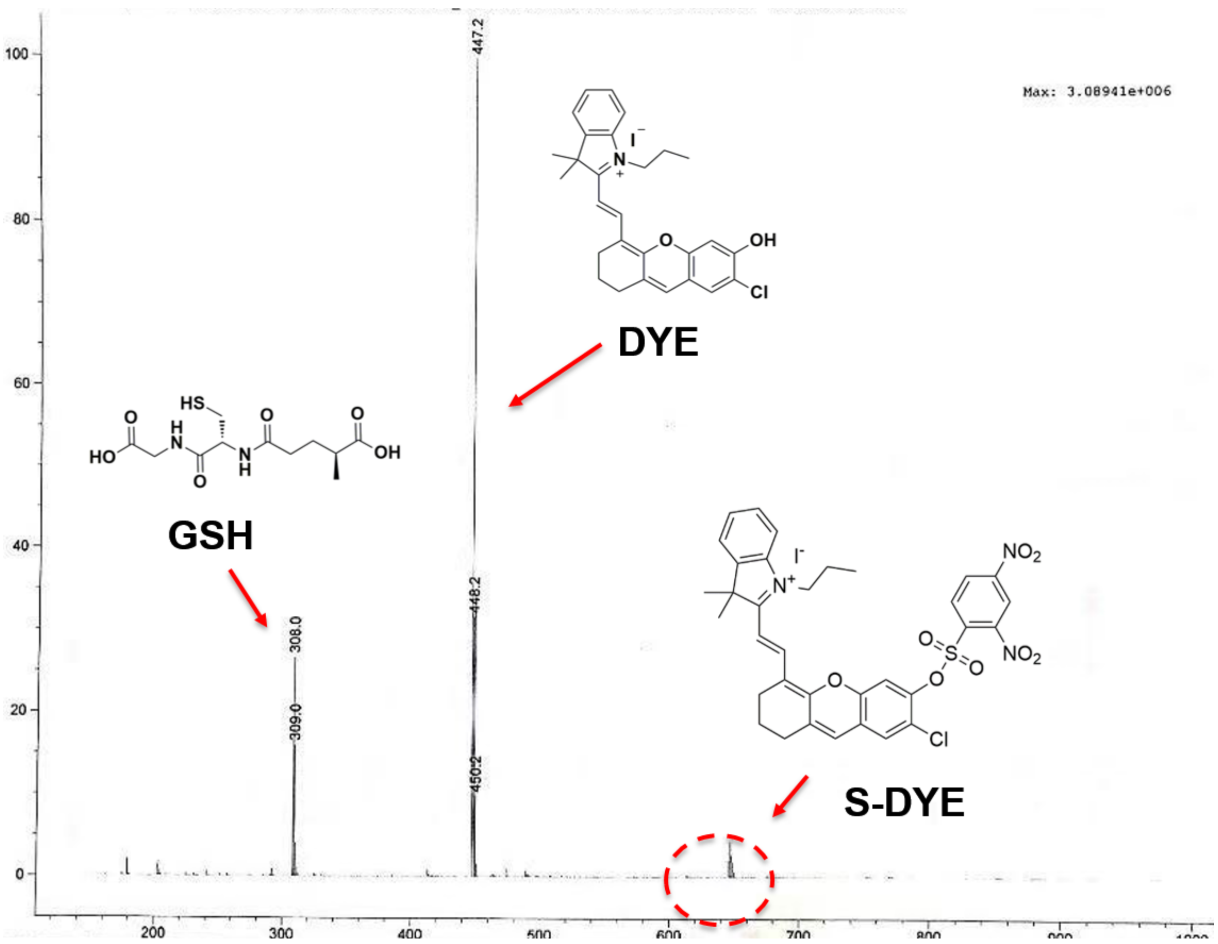

Fig. S21. A) The ESI-MS result of **S-DYE** upon being treated with GSH in mixed ACN:water (1:1, v:v, 37 °C) for 12 h, where only trace **S-DYE** was left, and free **DYE** was found liberated.

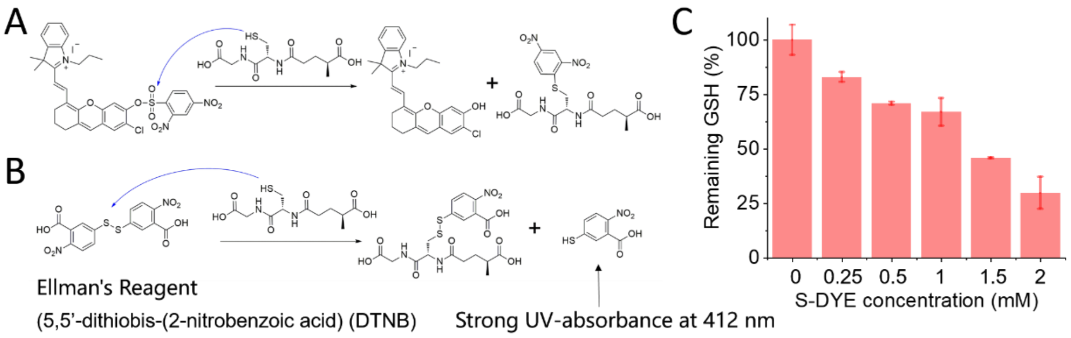

Fig. S22. Employing Ellman's Reagent (DTNB) to detect the left GSH after the 12-h consumption of **S-DYE** within GSH solution (2 mM, PBS 7.4). A) the detailed chemical reaction of GSH consumption by **S-DYE**; B) the intrinsic reaction of DTNB to the remaining GSH from the **S-DYE** 12-h consumption; C) the remaining GSH percentage after the 12-h consumption of **S-DYE** within GSH solution (2 mM, PBS 7.4).

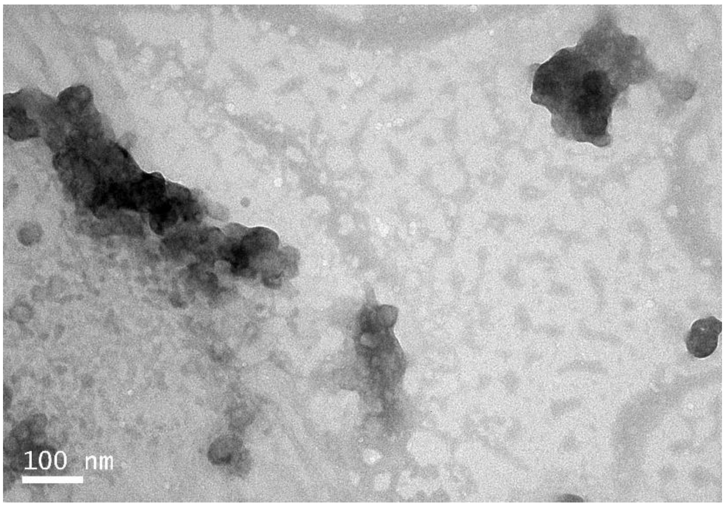

Fig. S23. Irregular micro-aggregate residue observed by TEM upon VC treatment.

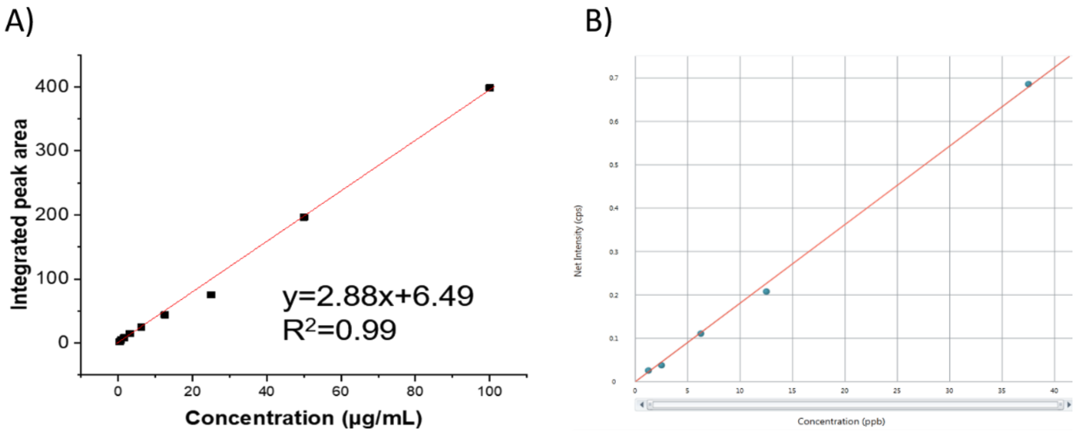

Fig. S24. Standard curve of oxaliplatin obtained from (A) HPLC with a UV-vis detector or (B) ICP-AES for the *in vivo* evaluation.

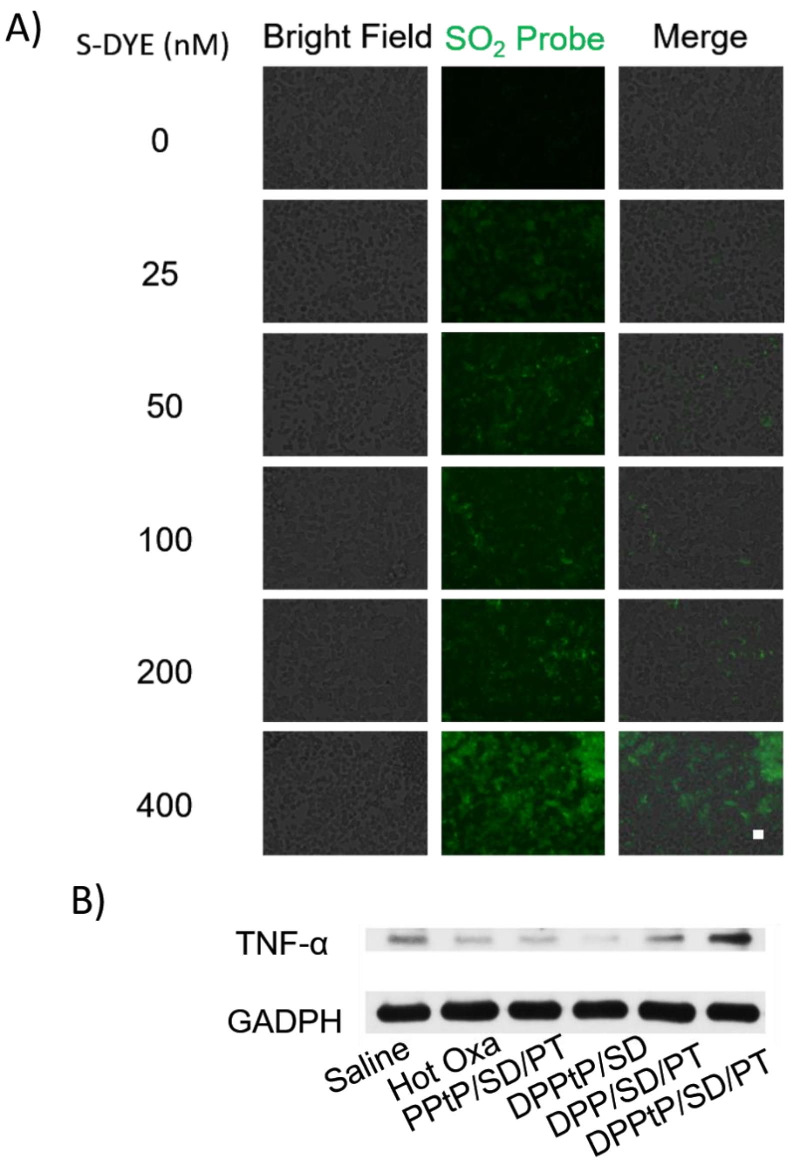

Fig. S25. Evaluation on the uncaged SO_2. A) The concentration dependence of the ROS level in relation with the addition with different amount of S-DYE on HCT-116 cells monitored by florescence microscopy (scale bar indicates 100 μm); B) Western-Blots results on TNF-α expression of the tumors isolated from the orthotopic tumor-bearing mice post different treatment.

5. Tumor tracking

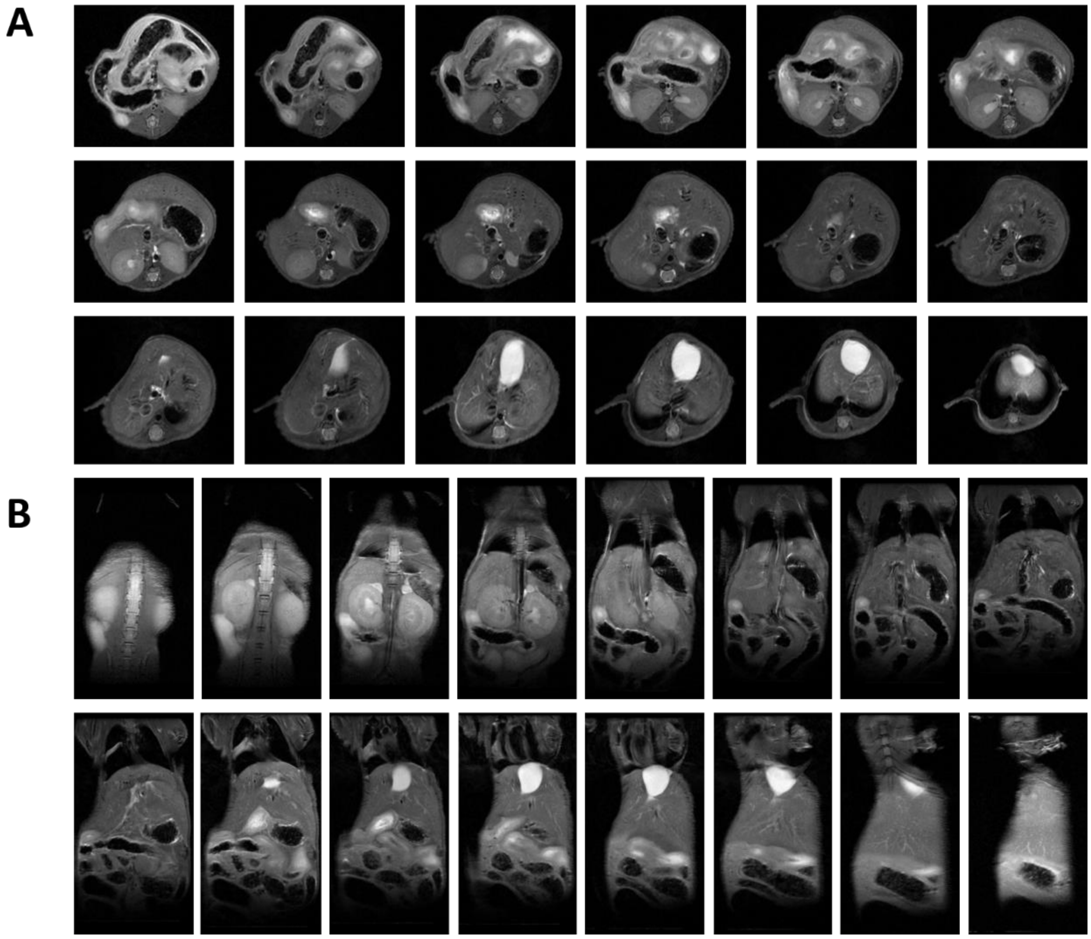

Fig. S26. Employing MRI to diagnose the orthotopic tumor-bearing mice: A) cross section; B) vertical section.

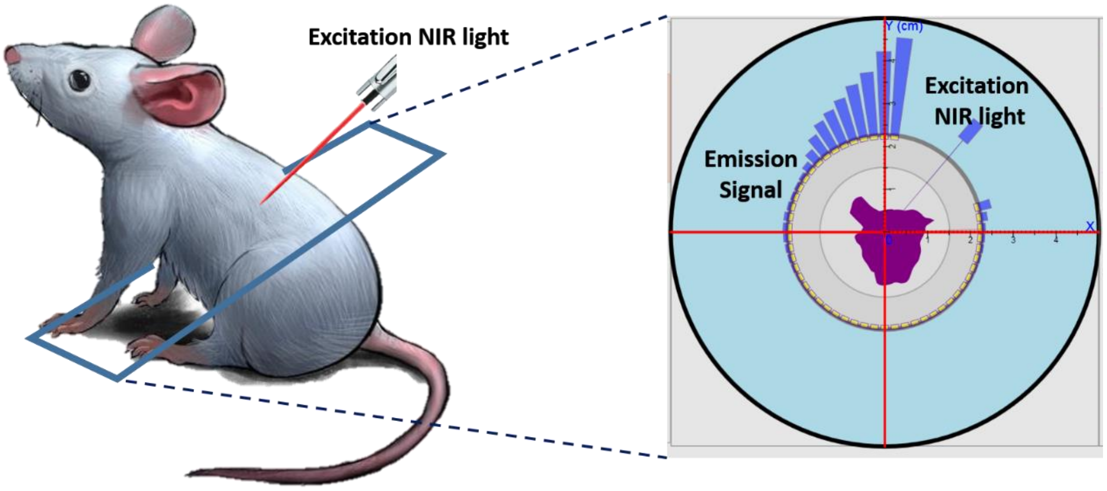

Fig. S27. Employing 3D-FLECT technique to characterize the orthotopic tumor-bearing mice at cross sections, where the emission signal indicates the suspected metastatic lesions. 3D *in vivo* fluorescence imaging and the film were taken from the FLECT/CT system (InSyTe™, TriFoil Imaging, www.trifoilimaging.com, Chatsworth, CA, USA).

Entry No.	Fluorescence signal	Photoacoustic imaging	Bioluminescence signal	Anatomical findings	Synergetic diagnosis
M1	+	+	+	+	
M2	+	+	+	+	
M3	-	+	+	+	
M4	+	+	+	-	
M5	-	-	-	-	
M6	+	+	+	+	
M7	+	+	-	-	
M8	+	+	+	+	
M9	+	-	-	-	
M10	-	-	+	+	

Table S4. Synergetic tumor verdicting results of the orthotopic tumor-bearing mice (14 days post tumor implantation) from different detection means. In this diagram, deep green indicates +2 precision, light green indicates +1 precision, deep red indicates +2 roughness, and light red indicates +1 roughness. The precision was confirmed after comparing with the anatomical and HE results.

6. Photothermal property

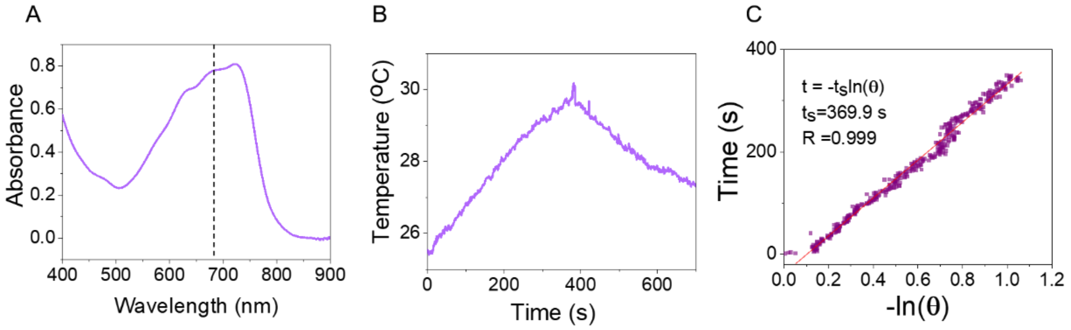

Fig. S28. A) The UV-absorbance of the formulated DYE in the aqueous solution; B) the heating-cooling curves of the formulated DYE in the aqueous solution irradiated with a 680-nm laser (0.3 W/cm^2); C) the τ_s value obtained from recording the heating-cooling curves.

The calculation of the photothermal conversion efficiency

The UV-absorbance value of the formulated DYE in the aqueous solution at 680 nm was measured to be 0.77454 as shown in Fig. S28A. A 680-nm laser (0.3 W/cm^2) was used to calculate the photothermal conversion efficiency. As shown in Fig. S28B, the temperature rose from 25.5 to 31.2 ^{o}C in ca. 400 s, while there was nearly no temperature change in the solution without the formulated DYE upon the same laser irradiation. Then the laser was shut down to allow the cooling procedure, and the cooling curve was recorded.

The total energy conversion during the photothermal procedure can be calculated from the below formula:

$$\sum_i m_i C_{p,i} \frac{dT}{dt} = Q_{cs} + Q_B - Q_{sur} \qquad (1)$$

where *m* represents the solvent's mass, C_p is the specific heat capacity, *T* is the solution temperature, Q_{cs} is the energy generated from the system, Q_B is the thermal energy generated from the vial, Q_{sur} is the released thermal energy into the air during the heat conduction. Then,

$$Q_{cs} = I(1 - 10^{-A_{680}})\eta \qquad (2)$$

where *I* is the laser power, η is the photothermal conversion efficiency,

A_{680} is the UV-absorbance value at 680 nm, Q_B is the dissipative enery from the vial absorbance during the measurement. Notbly, there was nearly no temperature change in the solution without the formulated DYE upon the same laser irradiation, and Q_B was ignored.

Q_{sur} is was directly proportional to the temperature,

$$Q_{sur} = hS(T - T_{\mathrm{amb}}) \qquad (3)$$

H is the efficiency constant of heat transfer, *S* is the superficial area of the vial, T_{amb} is the environment temperature.

When the system reaches the maximum value, the input heat energy equals to the output energy,

$$Q_{CS} + Q_B = hS(T_{max} - T_{amb}) \tag{4}$$

Therefore, the photothermal conversion efficiency can be calculated from the below formula:

$$\eta = \frac{hS(T_{max} - T_{amb}) - Q_B}{I(1 - 10^{-A_{680}})} \tag{5}$$

where, Q_B was measured to be 0 mW. Based on the heating curve upon laser-irradiation from the results of Fig. S28B, (T_{max}-T_{amb}) is 4.7 ^{o}C, *I* is 0.3 W/cm^2, and A_{680} is 0.77454. *hS* was calculated by introduction with θ, with a defination as:

$$\theta = \frac{T - T_{amb}}{T_{max} - T_{amb}} \tag{6}$$

The time constant τ_s of the sample can be defined as:

$$\tau_s = \frac{\sum_i m_i C_{p,i}}{hs} \tag{7}$$

Which as assigned into formular (1),

$$\frac{d\theta}{dt} = \frac{1}{\tau_s}\left[\frac{Q_{CS} + Q_B}{hS(T_{max} - T_{amb})} - \theta\right] \tag{8}$$

During the photothermal conversion, and the laser was shot down to result Q_{CS} +Q_B = 0, and the formula was simplified to give:

$$dt = -\tau_s \frac{d\theta}{\theta} \tag{9}$$

which was integrated as:

$$t = -\tau_s \ln\theta \tag{10}$$

Thus, during the cooling procedure of the sample, the fitting analysis was carried out to give the heat conduction constant τ_s as 369.9 s, and *m* is 1 g, *C* is 4.2 J/g.

Based on the formular (7), hS is 11.4 mW/°C, which was assigned into formula (5) to give the η value as 21.5 %.

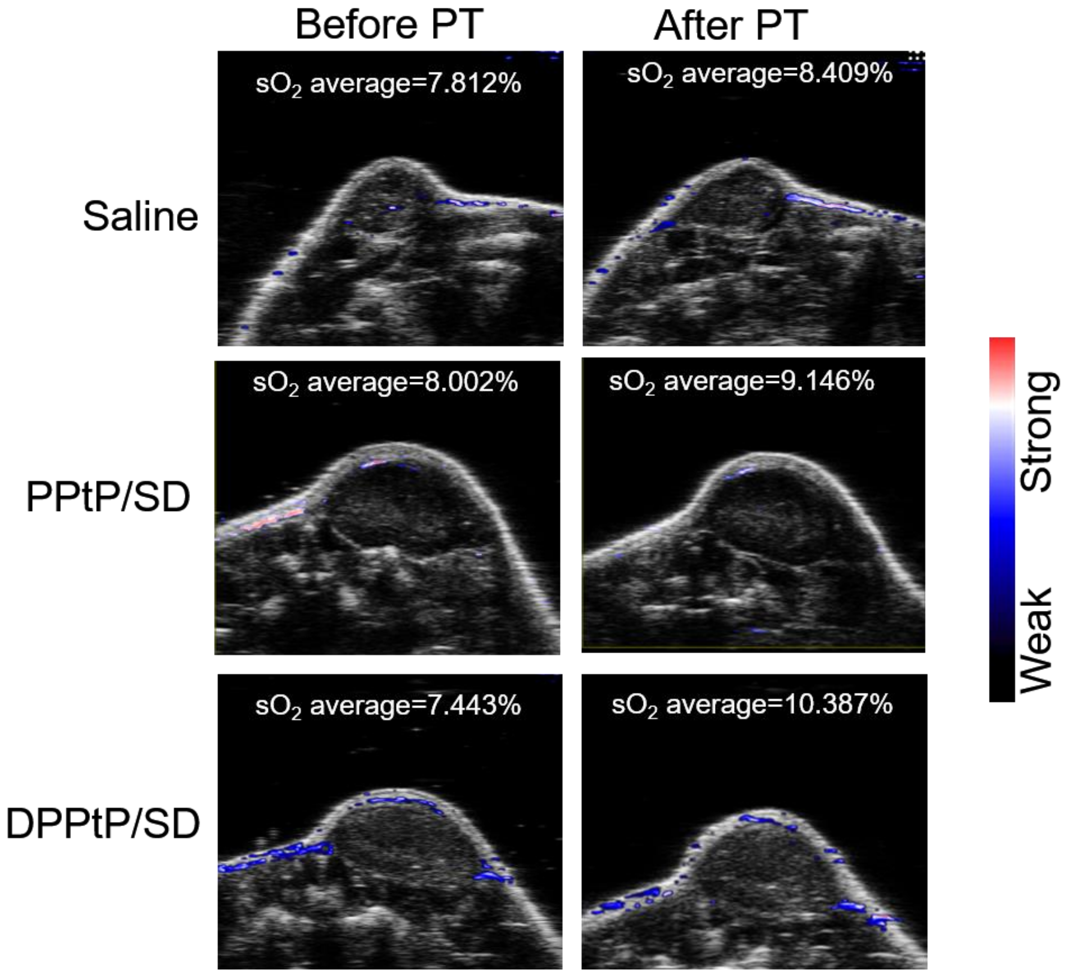

Fig. S29. The blood oxygen content increase upon a 680-nm laser irradiation (0.3 W/cm^2, 5 min) evaluated on the subcutaneous tumor-bearing mice by photoacoustic imaging.

7. Antitumor efficacy

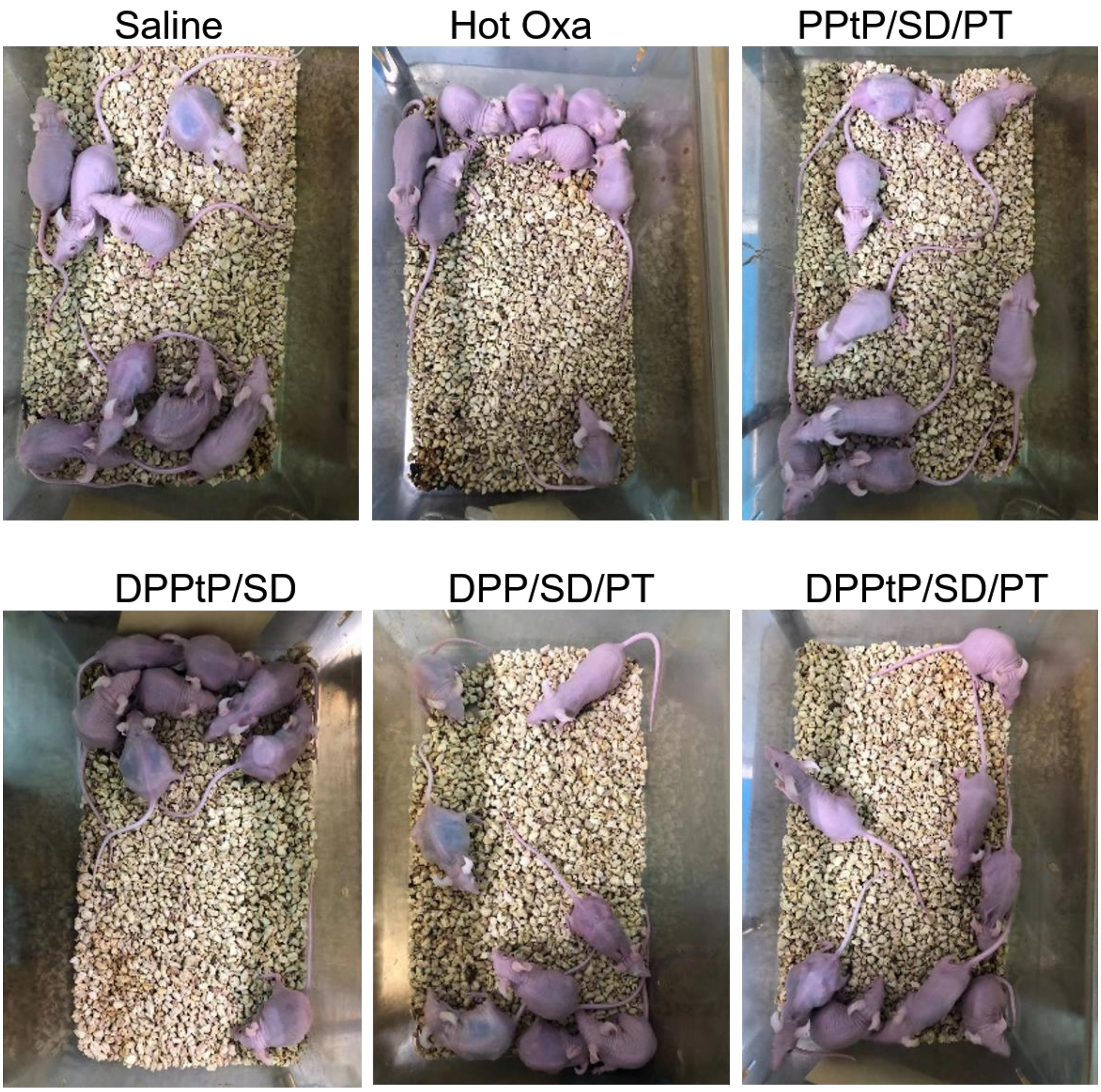

Fig. S30. The living conditions of the orthotopic tumor-bearing mice after receiving thrice treatment with different formulations.

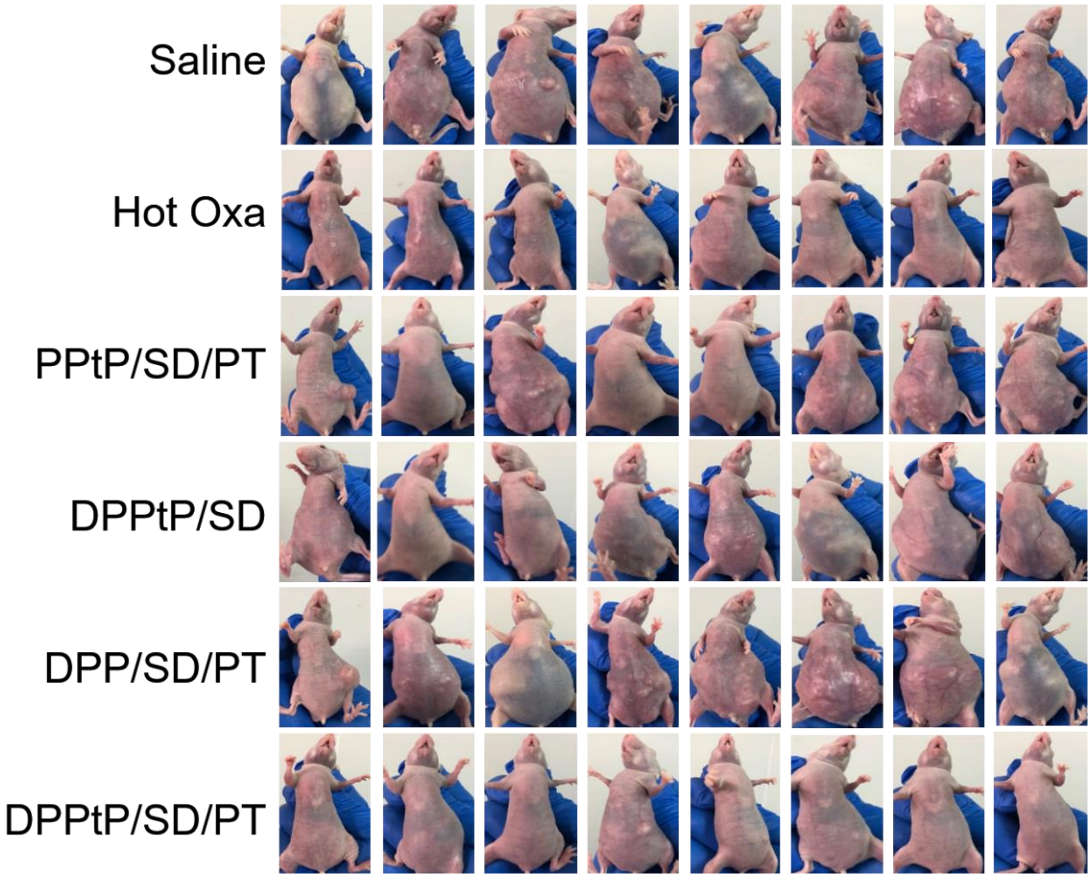

Fig. S31. Images of the orthotopic tumor-bearing mice after receiving thrice treatment with different formulations.

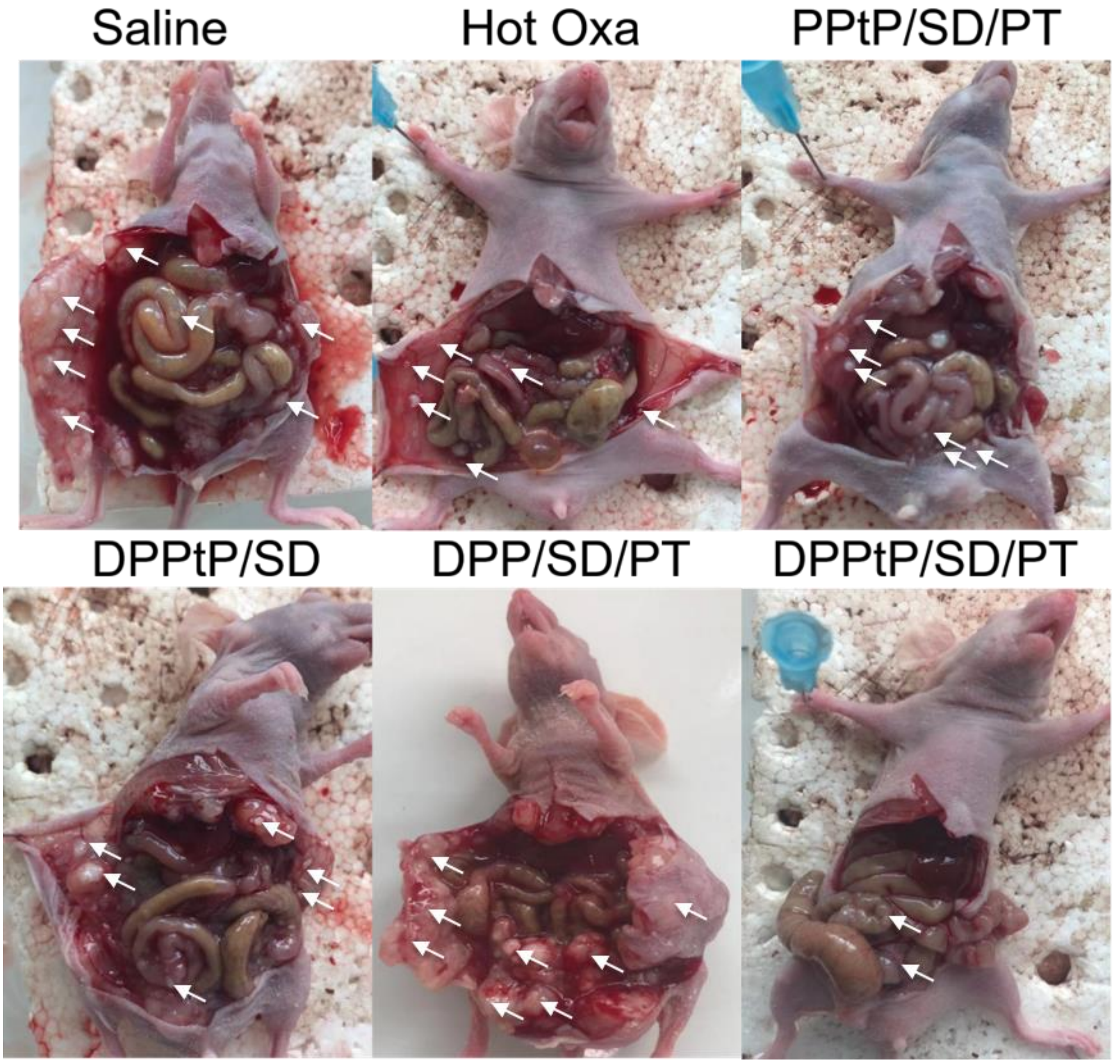

Fig. S32. Representative images of the dissected orthotopic tumor-bearing mice after receiving treatment with different formulations, where the arrows indicate the visible tumor niches.

8. References

1. Sun, T. et al. A targeting theranostics nanomedicine as an alternative approach for hyperthermia perfusion. *Biomaterials* **183**, 268–279 (2018).
2. Zhang, J. et al. Naked-eye and near-infrared fluorescence probe for hydrazine and its applications in *in vitro* and *in vivo* bioimaging. *Anal. Chem.* **87**, 9101–9107 (2015).
3. Yang, Y. et al. A novel coumarin-based fluorescent probe for selective detection of bissulfite anions in water and sugar samples. *Sens. Actuators B Chem.* **166–167** 665–670 (2012).
4. Sun, T. et al. Pre-blocked molecular shuttle as an *in-situ* real-time theranostics. *Biomaterials* **204**, 46–58 (2019).
